# Trends and predictions in demographic structures and metabolic health challenges for Chinese children and adolescents: insights from multiple waves of national school health surveys from 2000 to 2030

**DOI:** 10.1016/S2352-4642(25)00140-3

**Published:** 2025-06-19

**Authors:** Xinli Song, Bin Zhou, Sarah Baird, Chunling Lu, Zhiying Song, Yi Zhang, Ruolin Wang, Jianuo Jiang, Li Chen, Jieyu Liu, Wen Yuan, Yunfei Liu, Jiajia Dang, Peijin Hu, Jun Ma, Yanhui Dong, Yi Song, Majid Ezzati, Susan M Sawyer

**Affiliations:** 1 Institute of Child and Adolescent Health & School of Public Health, Peking University; National Health Commission Key Laboratory of Reproductive Health, Beijing, China; 2 School of Public Health, Imperial College London, London, UK; 3 Department of Global Health, Milken Institute School of Public Health, George Washington University, Washington DC, USA; 4 Division of Global Health Equity, Brigham and Women's Hospital, Boston, Massachusetts, USA; 5 Centre for Adolescent Health, Royal Children's Hospital, Parkville, Victoria, Australia; 6 The University of Melbourne, Faculty of Medicine, Dentistry and Health Sciences, Department of Paediatrics, Parkville, Victoria, Australia; 7 Murdoch Children's Research Institute, Parkville, Victoria, Australia

**Keywords:** Excess body weight, Obesity, Child and adolescent population, Hypertension, Metabolic burden

## Abstract

**Background:**

Understanding the changing metabolic health burden among children and adolescents is critical for public health resource allocation, now and into the future, particularly given the rapid ageing of the population in China. However, comprehensive studies characterizing these trends at national and provincial levels are lacking. This study fills this gap by estimating trends in the metabolic burden in children and adolescents from 2000 to 2030, using overweight and obesity (OWOB) and hypertension (HTN) as proxy indicators of metabolic burden.

**Methods:**

Anthropometric measurements from 1,106,416 children and adolescents aged 7 to 18 were extracted from five cycles of the Chinese National Surveys on Students Constitution and Health (2000, 2005, 2010, 2014, 2019). Nutritional status was defined using 2000 U.S. CDC criteria and HTN and severe HTN were defined using 2017 AAP criteria. Multinomial regressions were applied to project age-specific and sex-specific prevalence rates for one-year age groups in 2030. The Population Development Index (PDI) was calculated as the natural log of (birth rate/death rate) × (population aged 0-14/population aged 65+). The decomposition method sourced from the Global Burden of Disease study was utilized to evaluate the individual contributions of three factors (age-specific prevalence, age distribution, and population growth) to the net change in case numbers.

**Findings:**

From 2000 to 2030, the. 7-18-year-old population is projected to decline by 95 million (34.4%), while OWOB, OWOB with HTN, severe obesity, and severe HTN cases are expected to rise by 39.0 million (180.6%), 7.1 million (131.5%), 4.3 million (430.0%), and 1.2 million (34.3%), respectively. HTN cases remain stable (-0.8%). In 2019, the age-standardized prevalence rates were as follows: OWOB at 21.5% (95% CI: 21.3–21.7), HTN at 16.6% (95% CI: 16.4–16.8), OWOB with HTN at 5.5% (95% CI: 5.4–5.6), severe OB at 1.6% (95% CI: 1.5–1.6), and severe HTN at 2.1% (95% CI: 2.0–2.2). Significant negative associations were observed between the PDI and metabolic risk in both 2019 (*r* = -0.485, *p* = 0.006) and 2030 (*r* = -0.417, *p* = 0.020). Decomposition analysis indicates rising prevalence as the primary driver of case increases, partially offset by population decline.

**Interpretation:**

In the context of China's rapid demographic transition, the decline in youth populations contrasts with escalating prevalence, clinical severity, and absolute case numbers of OWOB and HTN, signaling a worsening metabolic health burden. Beyond public health policies to shape healthier patterns of physical activity, screen time and food, addressing these challenges will require optimizing the allocation of pediatric healthcare resources and enhancing efforts to improve the quality of China's primary healthcare system.

**Funding:**

The present study was supported by the National Natural Science Foundation of China (82103865 and 82373593 to YD; 82273654 to YS), Beijing Natural Science Foundation (7222244 to YD; 7222247 to YS), Peking University Talent Introduction Program Project (BMU2023YJ011 to YD), and Clinical Medicine Plus X-Young Scholars Project of Peking University (PKU2023LCXQ042 to YD). BZ and ME are supported by a grant from the UK Medical Research Council (grant number MR/V034057/1). BZ is supported by a fellowship from the Abdul Latif Jameel Institute for Disease and Emergency Analytics at Imperial College London, funded by a donation from Community Jameel.

## Introduction

The world is undergoing a series of rapid demographic transitions. In countries in Northern Europe and East Asia, the impact is in an ageing population and the growing burden of chronic diseases.^1^ While many low- and middle-income countries (LIMICs), particularly in Africa and the Middle East, face growing youth populations, China's older population is ageing and its child and adolescent population has also shrunk dramatically, reflecting a decline in the country's total fertility rate to 1.15 in 2021.^2^ Notably, projections indicate that by 2024, overweight and obesity (OWOB) and hypertension (HTN) - paramount metabolic risks - will rank as leading causes of years-of-life lost among the top 20 global risk factors.^3,4^

Surveys such as the China Health and Nutrition Survey (CHNS) and the Chinese National Surveys on Students' Constitution and Health (CNSSCH) have documented the prevalence and long-term trends of OWOB and HTN among Chinese children and adolescents at the national level.^5–7^ However significant data gaps remain at the provincial level, and around future projections. Additionally, there is currently no reliable and nationally representative data on the prevalence of severe obesity, severe HTN, or the coexistence of OWOB and HTN in this population. These children and adolescents face elevated cardiovascular risks in adulthood, along with the potential economic burdens that could stem from pharmacological or surgical interventions for those most affected.^8^ Furthermore, although metabolic risks such as OWOB and HTN are rising overall, the declining child and adolescent population raises critical questions about whether the absolute metabolic burden in children and adolescents is increasing, decreasing, or remaining stable. In the context where pediatric and geriatric healthcare development are equally important,^9^ understanding the provincial distribution of pediatric metabolic burdens in relation to demographic characteristics could guide policymakers in optimizing resource allocation.

To address these knowledge gaps, the present study is designed to: 1) undertake a thorough and comprehensive analysis, coupled with long-term projections, of the demographic shifts among Chinese children and adolescents aged 7 to 18 years from 2000 to 2030, juxtaposed against the secular trends and future estimates of the relative and absolute burdens of metabolic diseases, encompassing the prevalence and case counts of OWOB, HTN, OWOB comorbid with HTN, severe obesity, and severe HTN, at both the national and provincial levels; and 2) explore the evolving relationship between intra-provincial variations in demographic characteristics, such as birth rate, death rate, the proportion of children aged 0-14, and the proportion of individuals aged 65 and above, and the distribution of pediatric metabolic burdens across the nation. By doing so, this study aims to provide forward-looking evidence for the formulation of preventive strategies for metabolic diseases among children and adolescents that are tailored to demographic changes, as well as for the precise allocation of healthcare resources.

## Methods

### Study Population and Data Source

Data on age, sex, measured height (cm), weight (kg), and systolic and diastolic blood pressure (mmHg) for Han children aged 7–18 were obtained from five national cross-sectional surveys conducted in 2000, 2005, 2010, 2014, and 2019 as part of the CNSSCH. Covering 31 provinces in mainland China (excluding Hong Kong and Macau), the CNSSCH is the largest nationally representative survey of school-aged children. Sampling and data collection procedures were consistent across all survey years, as described previously.^10,11^ Briefly, a multistage stratified cluster sampling method was used. Each province was treated as an independent unit, and local school health institutes collected province-wide samples. Within each province, participants were stratified by sex and region (urban/rural), and further grouped by sex and age. Within each subgroup, classes were randomly selected from grades 1-12 in randomly chosen primary and secondary schools. The study was approved by the Medical Research Ethics Committee of Peking University Health Science Center (IRB00001052-18002), and verbal informed consent was obtained from both students and parents. A total of 7,864 observations (0.7%) were excluded due to missing data or extreme values (>5 SD from the sex- and age-specific mean across all years). The final analysis included 1,106,416 observations: 213,931 (2000), 236,532 (2005), 217,927 (2010), 216,764 (2014), and 221,262 (2019).

Data on the national age structure of China's population from 2000 to 2030, as well as national population data of 7-18-year-olds, were obtained from the United Nations.^12^ The proportion of the population aged 0-14 in each province to the national population aged 0-14 was obtained from the China Statistical Yearbook.^13^ We extrapolated this proportion with the national populations aged 7-18 to estimate the number of individuals aged 7-18 in each province; as these proportions were unavailable for 2030, we established estimates according to the hypothesis of unchanged distribution of the child population by province from 2019 to 2030. In addition, as no provincial age structure data were available, we applied the national unified age structure and province-specific population to estimate the province-level age-specific population aged 7-18. The Seventh National Population Census Bulletin reported demographic indicators such as aging rates and total fertility rates for each province in China in 2020, which we used to represent the demographic situation in 2019.^14^

### Definitions

#### Overweight, obesity and severe obesity

BMI was calculated as weight (kg) divided by height squared (m^2^), rounded to one decimal place. BMI categories were based on the CDC BMI-for-Age Growth Charts (2000):^15^ overweight (OW, 85^th^-95^th^ percentile), obesity (OB, ≥95^th^ percentile), and severe obesity (≥120% of the 95^th^ percentile or ≥35 kg/m^2^).

#### Hypertension, and severe hypertension

According to the Clinical Practice Guideline from the American Academy of Pediatrics (2017), ^16^ sex-, age-, and height-specific percentiles for systolic (SBP) and diastolic blood pressure (DBP) were calculated. HTN stages were defined separately for children aged 7-12 and adolescents aged 13-18. For children <13 years, Stage 1 HTN was defined as ⩾95th percentile to <95th percentile +12 mmHg, or 130/80 to 139/89 mmHg (whichever is lower); Stage 2 HTN (severe hypertension) as ⩾95th percentile +12 mmHg, or ⩾140/90 mmHg (whichever is lower). For those ⩾13 years, Stage 1 HTN was 130/80 to 139/89 mmHg; Stage 2 HTN (severe) was ⩾140/90 mmHg. Coexisting OWOB and HTN was defined as the presence of either overweight or obesity with HTN.

#### Population Development Index

Sex-specific death rate, fertility, and age-disaggregated population size (0-100+) were obtained from China's 2020 Seventh National Population Census. These data were used to build a cohort-component projection model to forecast the population in 2030. We estimated the proportion of the population aged 0-14 (%), aged 65+ (%), the birth rate (‰), and the death rate (‰) for that year to calculate the Population Development Index (PDI). The PDI was computed as the natural logarithm of: (birth rate / death rate) × (proportion aged 0–14 / proportion aged 65+). A PDI value below 0, especially the smaller it is, indicates a more inverted population pyramid. Details on anthropometric measurements and statistical analyses are provided in Supplementary Materials **(appendix pp. 35-42)**.

#### Role of the funders

The funders had no direct role in data collection, analysis, interpretation, writing of the manuscript, or the decision to submit.

## Results

### National demographic trends and metabolic health burden for Chinese children and adolescents

**Figure 1:**
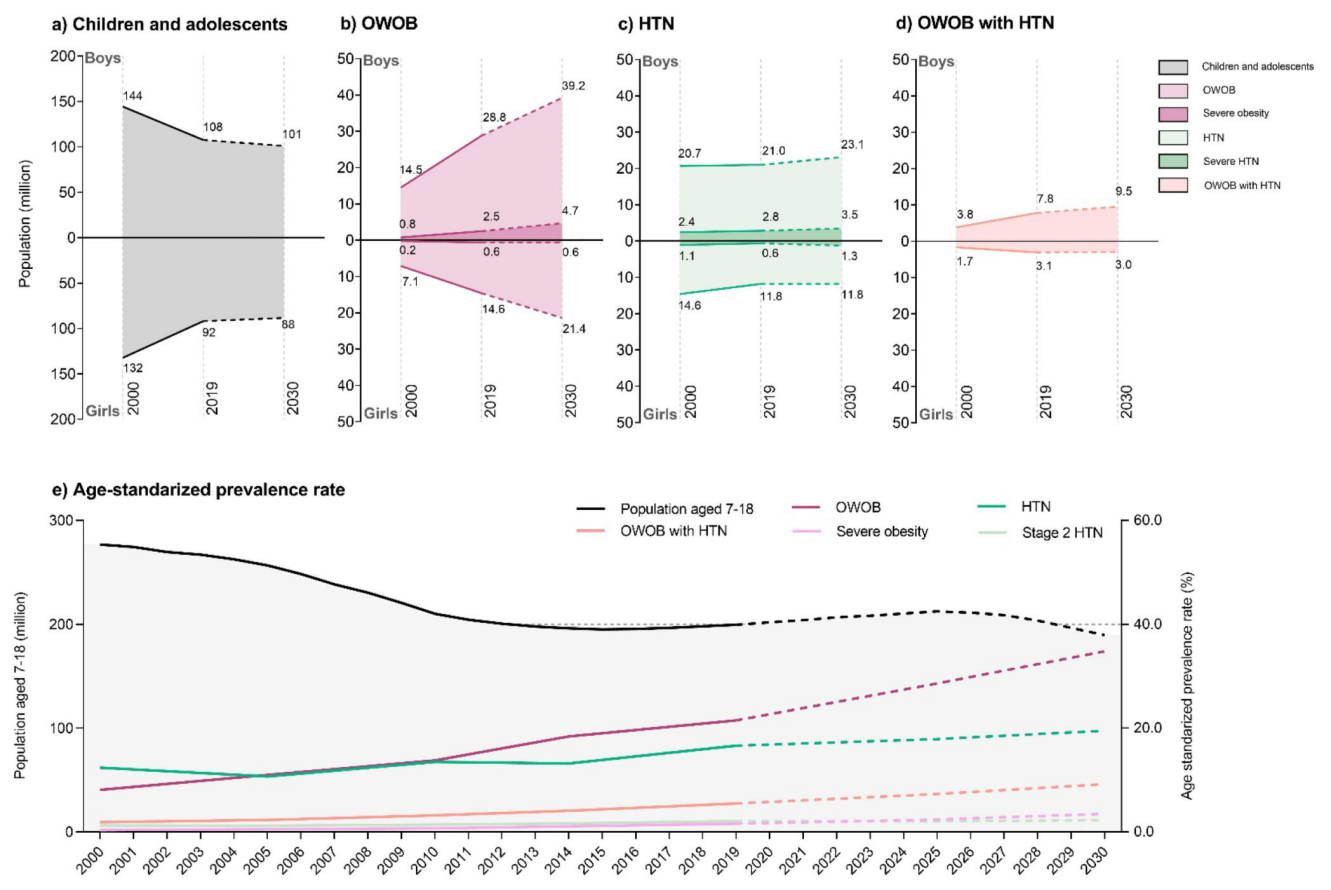
The number and age-standardized prevalence of OWOB, HTN, OWOB coexisting with HTN, severe OB, and severe HTN among Chinese children and adolescents aged 7-18 years, 2000-2030. (a) Population numbers (millions) of children and adolescents aged 7-18 years old, stratified by sex, in 2000, 2019, and 2030. The number of cases (millions) of (b) OWOB, and severe OB, (c) HTN, and stage 2 HTN, and (d) OWOB coexisting with HTN among Chinese children and adolescents, stratified by sex, in 2000, 2019, and 2030. (e) Age-standardized prevalence rates of OWOB, HTN, OWOB coexisting with HTN, severe OB, and stage 2 HTN. *Note*: OWOB = overweight and obesity; HTN = hypertension; OB = obesity. The age- and sex-specific standardized coefficients were based on the CNSSCH data in 2019. Source data are provided in the Supplementary files.

Over the past two decades, Chinese children and adolescents exhibited significant increases in height, weight, and BMI (*p* for trend <0.05, Table S1), while SBP and DBP showed only slight, non-significant increases (*p* for trend >0.05). The population declined from 276 million (2000) to 200 million (2019), and is projected to decrease further to 181 million by 2030 (–34.4%, Figure 1e). Age-standardized prevalence rates of OWOB, HTN, OWOB coexisting with HTN, severe OB, and severe HTN rose steadily (Figure 1e, Table S4) (**appendix p 7**). In 2019, the age-standardized prevalence rates for both sexes combined were 21.5% [21.3–21.7] for OWOB, 16.6% [16.3–16.9] for HTN, 5.5% [5.4–5.6] for OWOB coexisting with HTN, 1.57% [1.52–1.62] for severe OB, and 2.09% [2.03–2.15] for severe HTN. By 2030, these are projected to rise to 34.8% [34.3–35.3], 19.5% [19.1–19.9], 9.2% [9.0–9.4], 3.50% [3.16–3.84], and 2.27% [2.06–2.48], respectively. Considering population decline, absolute case estimates revealed the metabolic health burden (Figure 1, Table S3) (**appendix p 6**). The estimated number of OWOB cases rose from 21.6 million [95% CI 21.1–22.0] in 2000 to 43.4 million [95% CI 42.8–44.0] in 2019 and is projected to reach 60.6 million [95% CI 59.7–61.5] by 2030, reflecting an increase of 181% from 2000 to 2030 (see Figure 1b, Table S3). HTN cases declined from 35.3 million [34.7–35.8] (2000) to 32.8 million [32.3–33.3] (2019), projected to be 35.0 million [34.3–35.7] (2030; stable, Figure 1c, Table S3). Severe HTN cases increased from 3.5 million [3.3–3.6] (2000) to 4.0 million [3.9–4.2] (2019), projected to reach 4.7 million [4.3–5.1] (2030; +34%, Figure 1c, Table S3). OWOB coexisting with HTN cases rose from 5.4 million [5.2–5.7] (2000) to 10.9 million [10.7–11.2] (2019), projected to reach 12.5 million [12.2–12.8] (2030; +131%, Figure 1d, Table S3).

### Provincial demographic trends and metabolic health burden for Chinese children and adolescents

**Figure 2:**
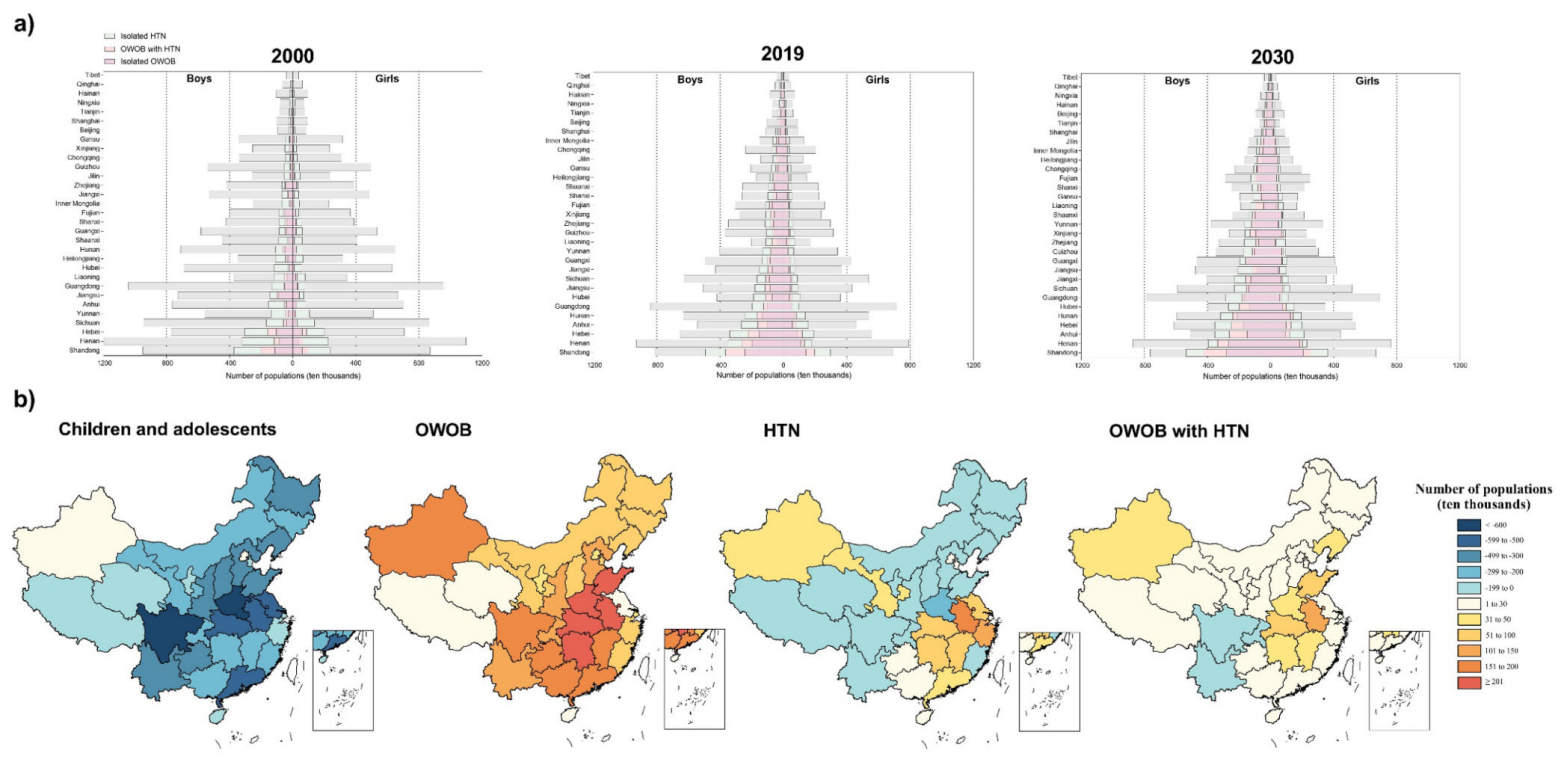
Province-specific numbers of cases of children aged 7-18 years old with OWOB, HTN and OWOB coexisting with HTN in 2000, 2019 and 2030. In Figure 2 (a), green, purple, light orange, and gray bars represent isolated HTN, isolated OWOB, OWOB coexisting with HTN, and populations without OWOB or HTN, respectively. *Note*: OWOB = overweight and obesity; HTN = hypertension. Source data are provided in the Supplementary files.

Figure 2a depicts the distributions of children and adolescents aged 7-18, isolated OWOB, isolated HTN, and OWOB with HTN cases for 2000, 2019, with projections for 2030, along with their provincial rankings. Observations align with national trends: the population of children and adolescents is declining, while their metabolic health burden (OWOB and HTN cases) is increasing. From 2000 to 2030, Shandong consistently bears the highest burden, followed by Henan. In Shandong, the population of children and adolescents decreased from 1.8 million in 2000 to 1.4 million in 2030. Concurrently, OWOB cases were 0.22 million [95% CI 0.20–0.25], HTN cases 0.44 million [95% CI 0.42–0.46], and OWOB with HTN cases 0.13 million [95% CI 0.12–0.14] in 2000 (Table S6) (**appendix p 15-19**). These are projected to increase to 0.72 million [95% CI 0.68–0.76], 0.36 million [95% CI 0.33–0.40], and 0.18 million [95% CI 0.16–0.19] by 2030 (Table S6).

Considering OWOB, HTN, and OWOB with HTN separately, substantial heterogeneity in case numbers and prevalence rates from 2000 to 2030 is evident across China's 31 provinces (Tables S5, S6). The population of children and adolescents is declining in nearly all provinces, with slight increases only in Beijing and Xinjiang. OWOB cases will increase variably, while HTN cases will rise in nearly half of the provinces (and decrease in others). OWOB with HTN cases will rise in most provinces, except Yunnan, Tibet, and Chongqing (Figure 2b). Contrary to the nationwide increase from 2000 to 2030, the age-standardized prevalence of HTN and OWOB with HTN in about half of the provinces and certain individual provinces is projected to decrease (Table S5) (**appendix p 8-14**).

### Population development index and metabolic risk for children and adolescents within country

Table S2 presents Chinese demographic trends in 2000, 2019, and 2030 at national and provincial levels (**appendix p 8-14**). National PDI levels were 2.00, 0.67, and -0.47 in 2000, 2019, and 2030, respectively, showing a continuous decline. Tibet ranked first nationwide for PDI in these years, with values of 3.29, 2.65, and 1.84. In 2000, Shanghai (-0.22) had the lowest PDI, while Liaoning (-0.56) and Heilongjiang (-2.11) held this position in 2019 and 2030, respectively. PDI is positively correlated with birth rates and the share of the population aged 0-14, and negatively correlated with death rates and the share of the population aged 65+ (Figures S1-S4).

**Figure 3:**
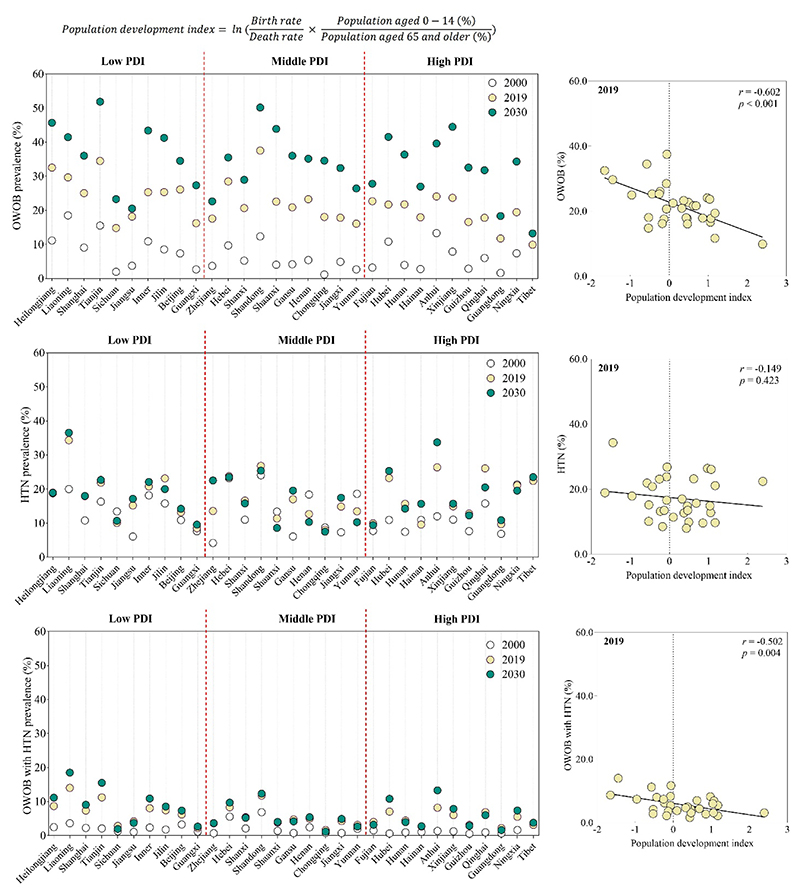
Provincial age-standardized rates (%) of OWOB, HTN, and OWOB with HTN among Chinese children and adolescents aged 7-18 in 2000, 2019, and 2030, categorized by PDI *Note*: The high, middle, and low PDI group are categorized based on the tertiles. OWOB = overweight and obesity; HTN = hypertension; PDI = population development index. Source data are provided in the Supplementary files

**Figure 4:**
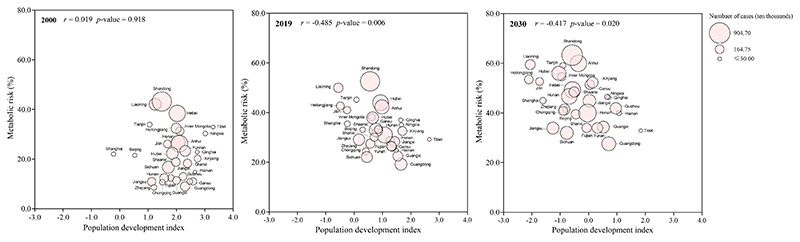
Associations of pediatric metabolic risk with population development index in China, in 2000. 2019, and 2030. *Note*: Pediatric metabolic risk was calculated by the sum of provincial age-standardized rates (%) of OWOB and HTN. OWOB = overweight and obesity; HTN = hypertension.

Figure 3 depicts age-standardized prevalence rates of OWOB, HTN, and OWOB with HTN in 2000, 2019, and 2030. In 2019, Shandong had the highest OWOB prevalence at 37.5% [95% CI 36.1–38.9]. By 2030, nine provinces are projected to exceed 40%, with two surpassing 50% (Figure 3, Table S5). For HTN, Liaoning recorded the highest prevalence at 34.3% [95% CI 32.9–35.8] in 2019, and by 2030, one province is projected to exceed 35% (Figure 3, Table S5). For OWOB with HTN, Liaoning had the highest prevalence at 14.0% [95% CI 13.1–14.9] in 2019, and by 2030, two provinces are projected to exceed 15% (Figure 3, Table S5). In 2019, provinces with lower PDI showed higher prevalence of OWOB (*r* = -0.472, *p* = 0.007) and OWOB with HTN (*r* = -0.502, *p* = 0.004), but no significant correlation with HTN (*r* = -0.149, *p* = 0.423). Figure 4 illustrates the association between PDI and pediatric metabolic risk (combined prevalence of OWOB and HTN) across provinces in 2000, 2019, and 2030. No significant association was observed in 2000 (*r* = 0.019, *p* = 0.918), but significant negative associations were found in 2019 (*r* = -0.485, *p* = 0.006) and 2030 (*r* = -0.417, *p* = 0.020).

### Decomposition

**Figure 5:**
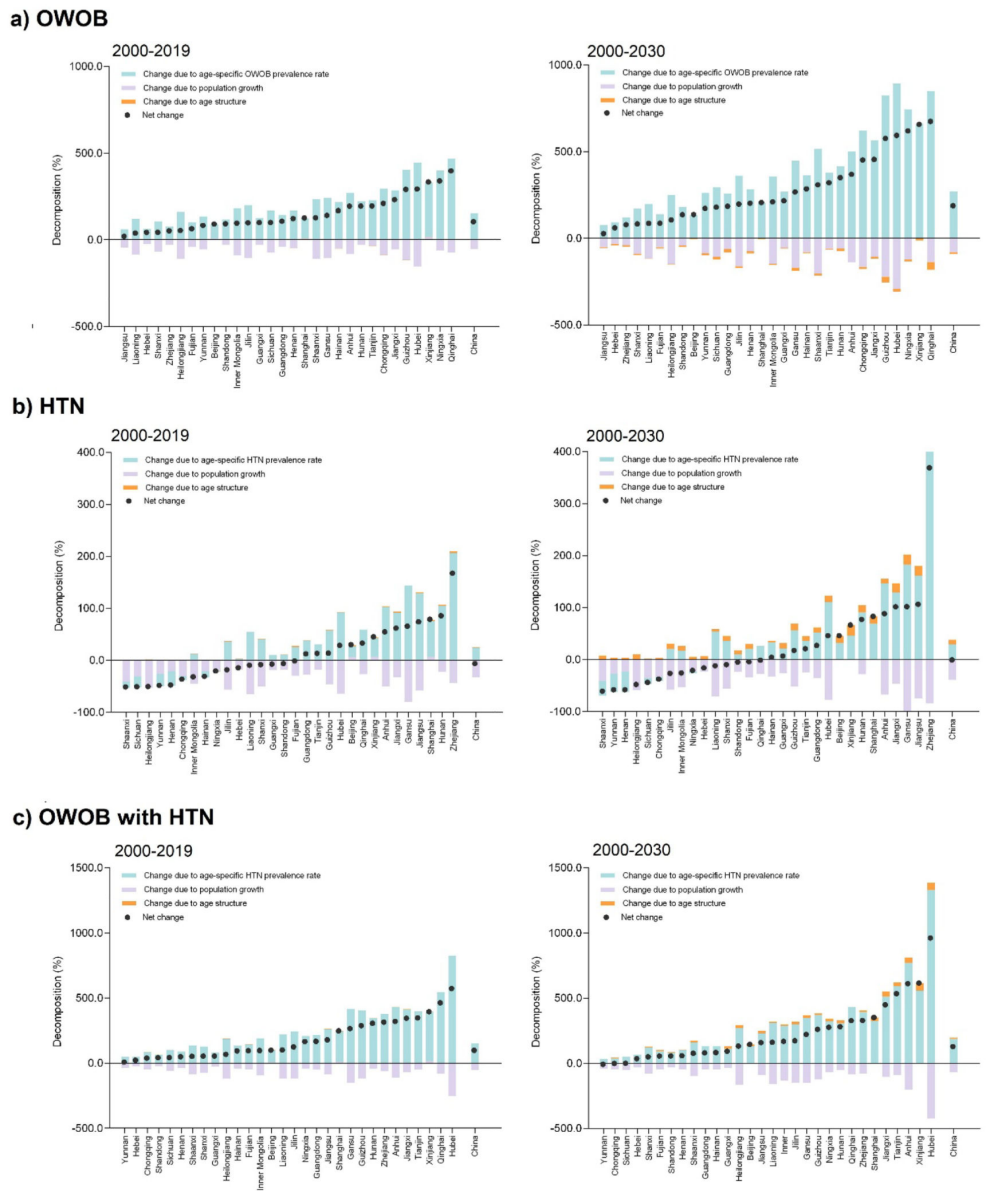
Decomposition of changes in (a) OWOB, (b) HBP and (c) OWOB with HTN cases attributable to population growth, age structure, and age-specific prevalence rates among Chinese children and adolescents aged 7 to 18, between 2000-2019 and 2000-2030 *Note*: OWOB = overweight and obesity; HTN = hypertension.

From 2000 to 2019, OWOB cases increased by 100.8%, driven primarily by rising age-specific prevalence rates (+152.2%) and changes in age distribution (+0.4%), which offset population declines (-51.8%) (Figure 5a). This trend is projected to intensify by 2030, with OWOB cases increasing by 180.5%, largely due to rising age-standardized prevalence rates (+270.9%), outweighing the negative impacts of declining child and adolescent populations (-81.2%) and age distribution (-9.2%) (Figure 5a). HTN cases remained relatively stable from 2000 to 2019 (-7.5%) and 2000 to 2030 (-1.3%), as rising prevalence rates (+24.4% and +29.4%, respectively) largely offset population declines (-32.6% and -38.9%, respectively) (Figure 5b). Additionally, OWOB with HTN cases increased by 100.0% from 2000 to 2019 and 129.1% from 2000 to 2030, primarily due to rising prevalence rates (+152.1% and +187.9%, respectively), which offset population declines (-52.5% and -68.8%) and age distribution changes (+0.4% and +10.0%) (Figure 5c).

## Discussion

This study is the first to comprehensively assess and project the absolute and relative metabolic health burdens, indicated by OWOB and HTN, among Chinese children and adolescents aged 7-18 from 2000 to 2030, using multiple waves of nationally representative, school-based data. Findings reveal a dual crisis, as a 34.4% reduction in population size is accompanied by a rapid escalation in metabolic health burdens, with cases of OWOB, OWOB with HTN, severe obesity, and severe HTN projected to rise by 180.6%, 131.5%, 430.0%, and 34.3%, respectively. Provinces with accelerated demographic transitions, marked by declining young populations and advanced aging, face disproportionately greater metabolic health challenges in children and adolescents. The concurrent trends of declining young populations, escalating metabolic burdens, and widening structural disparities highlight that child and adolescent metabolic health is an increasingly severe public health challenge in China in the post-COVID era.

In this study, the number of children and adolescents with OWOB is projected to increase from 43 million in 2019 to 60 million by 2030. This estimate is lower than the 68 million cases predicted by a meta-analysis^17^ and half as lower as 130 million cases projected in the World Obesity Federation's Obesity Atlas 2024.^18^ Our findings reveal that the rising prevalence of OWOB represents the primary driver of case number growth. In line with our findings, several projection studies consistently indicate that OWOB prevalence will continue its upward trajectory through 2030.^19^ While nationally representative severe obesity data remain limited in China, the epidemic follows established patterns where severe phenotypes rise proportionally with overall obesity. China's childhood obesity surge correlates with socioeconomic progress, potentially explained by increased household purchasing power, market-driven dietary changes, academic stress-related sedentariness, and public health governance gaps.^20^

Consistent with global data,^21^ a stark upward trend in HTN prevalence was observed in our study, from 12.4% in 2000 to 16.6% in 2019. While several national cross-sectional surveys, such as the China Child and Adolescent Cardiovascular Health Study 2013-2015 and the China Health and Nutrition Survey 2015, have reported higher prevalence estimates of HTN (ranging from 18.5% to 20.5%^7,22^), direct comparisons with these studies are limited by variation in age groups and diagnostic criteria. Nevertheless, HTN increases in Chinese youth are evident. The 2023 WHO Global Hypertension Report identifies population-level risk factors for HTN including high sodium and low potassium intake, alcohol consumption, physical inactivity, and air pollution.^23^ Recent evidence from a Chinese systematic review and modelling study demonstrates four significant predictors of elevated blood pressure in childhood: OWOB, salty food consumption, family history of hypertension, and physical inactivity.^24^

By focusing on the distribution of the metabolic burden in children and adolescents within the country, a concerning phenomenon we have identified that provinces with more pronounced demographic characteristics (lower birth rate/child proportion and higher death rate/elderly proportion) also face a heavier metabolic health risks. This phenomenon might be explained through the lens of China's growing urbanization since 1980, which is characterized by extensive industrialization and rapid socioeconomic growth.^25^ Similar to most countries worldwide, urbanization in China has been associated with population aging, a decline in fertility rates, and an increase in life expectancy. This is consistent with the statistically significant correlation between the higher urbanization rate and the lower PDI index observed in 2019 (see Figure S5) (**appendix p 32**). At the same time, urbanization also has a dual impact on child and adolescent health. On the one hand, rapid urbanization improves public services, including education and medical care, which has greatly improved child and adolescent health and well-being.^26^ On the other hand, urban environments promote food insecurity and air pollution, and changing lifestyles such as physical inactivity, and high intake of dietary fats and ultraprocessed foods^27^ which each promote child metabolic diseases. For example, data from the China Health and Nutrition Survey revealed that children and adolescents following a traditional Chinese dietary pattern (characterized by a high intake of rice, vegetables, poultry, pork, and fish) were less likely to be obese compared to those who followed a modern dietary pattern (characterized by a high intake of wheat, processed meats, and fast food).^28^ Further, modern dietary patterns have not led to a reduction in the Chinese population's preference for salt. A systematic review and meta-analysis in 2019 also found that Chinese children aged 3-6 years old consume the maximum amount of salt recommended by the WHO for adults (5g a day) while older children consume almost 9g/day.^29^ Furthermore, long-term exposure to PM2.5 increases the risk of metabolic disorders, including childhood OWOB and HTN.^30^

The escalating metabolic health burden among contemporary Chinese children and adolescents has emerged as a critical public health challenge with both immediate and long-term implications, necessitating the implementation of a comprehensive and coordinated national strategy. In addition to the first 1,000 days of life, cost-effective investments in interventions during the next 7,000 days, i.e., childhood and adolescence, can significantly enhance long-term health outcomes.^31^ Three key domains particularly stand out as needing attention - healthcare financing for adolescent populations, pediatric medical resource allocation, and delivery of essential public health services. Data from 2014 indicates that healthcare spending on adolescents in China accounted for only 2.6% of total national health expenditures, with per-adolescent health expenditure amounting to CNY 525 (USD 85.5), significantly lower than the per capita health expenditure of CNY 2349 (USD 382.4).^32^ Additionally, soaring absolute cases underscores the pressing need for pediatric medical resources and primary public health services. In fact, building on the existing tiered healthcare delivery system, China's pediatric healthcare system has long grappled with significant challenges, including unmet service demands, an imbalanced service structure, and inadequate pediatrician training.^33^ More recently, this has been exacerbated by growing demands for higher quality paediatric care, which likely reflects more educated and wealthy parents. Geographical disparity in the allocation of pediatric healthcare resources, juxtaposed against the urban-rural divide, will further exacerbate the multifaceted challenges confronting the healthcare system in mitigating the burgeoning metabolic burden, with concerns of growing inequity at multiple levels.^34^ Furthermore, the current pediatric service structure remains heavily dependent on tertiary hospitals, which presents substantial barriers to implementing the WHO-recommended obesity management framework that emphasizes the importance of primary health care.^35^ From a public health perspective, while a substantial proportion of children and adolescents require primary care interventions including lifestyle counseling and weight management, school-based and community health centers continue to suffer from severe resource inadequacies in China.^9^

One major contribution of this study spanning 30 years is the combined picture it provides of the demographic trends of population aging and declining fertility and increasing metabolic risks trends including OWOB and HTN among children and adolescents. To our knowledge it is the first Chinese study to measure trends in prevalence and case number estimates of severe obesity, and severe HTN, and OWOB coexisting with HTN, which are of major clinical as well as public health significance. We used decomposition analysis to quantify the effects of different drivers to derive more accurate and robust estimates, which allowed for a better understanding of the pediatric metabolic burden than other comparable epidemiologic research in China. Furthermore, we introduced the Population Development Index (PDI) as a novel indicator to capture demographic trends while simultaneously accounting for the influence of age structure. The PDI can be conceptualized as a social determinant of health, akin to socioeconomic development levels and urbanization rates, providing a holistic framework for assessing health disparities.

Our study also has several limitations. Firstly, due to data limitations, a unified national age structure was employed instead of individual provincial age structures, which may lead to an underestimation of the variations in case numbers among provinces for each of the years 2000, 2019, and 2030. Secondly, with only five surveys conducted between 2000 and 2019, the time-series data points for 2030 projections were limited. However, the performance of the prediction model for estimating the national prevalence of OWOB in 2019 using pre-2019 data appears to be relatively reliable: mean predictions across all age- and sex-specific groups remained within a 20% relative error of the reported estimates in 91.7% of cases, while achieving a mean absolute error of 2.32 percentage points. We utilized a substantial quantity of individual-level data points and generated uncertainty intervals for anticipated estimates through bootstrapping methods. Third, as the CNSSCH is a school-based survey, it excludes non-enrolled children and adolescents. Given the declining national dropout rate (junior secondary school: from 11.4% in 2000 to 0.5% in 2019), the concentration of early school leaving in rural and low socioeconomic status regions, and the documented lower metabolic health risks in these subgroups, our earlier estimates (e.g., 2000) may overrepresent true case numbers. Conversely, the 2019 estimates closely approximate actual values due to near-universal enrollment. This selection bias suggests that the true increase in China's metabolic health burden since 2000 may be marginally higher than our estimates.

In summary, leveraging authoritative and representative national data from China, this study has assessed the long-term trends and projections of demographic changes among children and adolescents, along with the corresponding relative and absolute burdens of metabolic diseases within this population. Despite a notable decrease in the population size of children and adolescents, the prevalence, severity, and absolute case counts of OWOB and HTN continue to escalate, underscoring the mounting pediatric metabolic burden both presently and in the foreseeable future. This alarming trend emphasizes the pressing need for high-quality pediatric healthcare resources and necessitates the establishment of a regionally balanced, primary health care-driven pediatric healthcare delivery system to optimize the health and well-being of affected children and adolescents. Furthermore, within the national context, provinces experiencing lower fertility rates and heightened population aging shoulder a disproportionately heavier pediatric metabolic burden. These regions are poised to be the first to confront the dual public health challenges posed by both elderly and young populations in China, a trend that holds the potential to spread across the entire country. To effectively address these multifaceted challenges, it is imperative to augment healthcare financing specifically tailored to children and adolescents, delineate the roles and responsibilities of key stakeholders—encompassing government entities, community organizations, educational institutions, and healthcare facilities—and foster collaborative efforts to promote the metabolic health of children and adolescents in China.

## Supplementary Material

appendix

## Data Availability

All data in this article can be shared. Requests with appropriate ethics board approvals and study protocols will be assessed by the Institute of Child and Adolescent Health, Peking University.

## Research in context

### Evidence Before this Study

We searched PubMed on December 20th, 2024 using the broad terms ("population" OR "demographics") AND ("metabolic health" OR "excess body weight" OR "overweight" OR "obesity" OR "overweight and obesity" OR "body mass index" OR "BMI") AND ("projection" OR "prediction" OR "forecast" OR "trend analysis" OR "modeling") AND ("metabolic health" OR "hypertension" OR "high blood pressure" OR "elevated blood pressure" OR "cardiovascular risk" OR "diabetes" OR "insulin resistance") AND ("trends" OR "patterns" OR "changes over time") AND ("Chinese" OR "China" OR "Mainland China" OR "Chinese population") AND ("youth" OR "children" OR "adolescents" OR "teenagers" OR "students" OR "school-aged children") for articles published in English. Previous studies, such as the China Health and Nutrition Survey (CHNS) from 1991 to 2015 and the Chinese National Surveys on Students Constitution and Health (CNSSCH) from 1995 to 2014, have reported changes in the prevalence of overweight and obesity (OWOB), and hypertension (HTN) among Chinese youth at the national or provincial level, but have not estimated the prevalence of severe obesity, severe HTN, nor the coexistence of OWOB with HTN. Additionally, data on the absolute burden and trends of children and adolescents affected by OWOB and HTN is limited but warrants attention, particularly in the context of low fertility rates. China's total population of children and adolescents remains high yet is rapidly decreasing. As the country is projected to enter an era of moderate to severe ageing and declining youth populations within the next decade, the knowledge gap around the absolute and relative metabolic burden of OWOB in young people is apparent. This understanding will guide future investments in health services with the opportunity to profoundly influence overall public health policies.

### Added value of this study

The particular value of this study lies in its revealing projections of the metabolic burden of overweight and obesity in children and adolescents from 2000 to 2030 in China. Despite projections of a dramatic 34.4% reduction in the population size of this demographic, there will concurrently be an alarming surge in the metabolic health burden. The number of cases of pediatric OWOB and HTN is anticipated to climb from 57 million to 95 million, with cases of severe obesity, severe HTN, and coexisting OWOB and HTN projected to soar by 430%, 34%, and 131%, respectively. Alarmingly, provinces with more pronounced characteristics of lower birth rate/child proportion and higher death rate/elderly proportion also face heavier pediatric metabolic burden.

These provinces are at the forefront of confronting dual public health challenges from both the elderly and children—a trend that is anticipated to spread nationwide. This necessitates a more precise allocation of medical and healthcare resources to address these disease burdens effectively, taking into account the unique challenges posed by each province's demographic profile.

### Implications of all the available evidence

Despite the declining population of Chinese children and adolescents, the metabolic burden on this group is steadily increasing, with the prevalence and severity of OWOB and HTN continuing to rise. Amid China's rapid demographic shifts, provinces with more pronounced aging and low birth rates are facing elevated pediatric metabolic risks, highlighting the urgent need for the government to balance healthcare priorities between the elderly and younger generations. To address this hidden crisis and reduce the healthcare burden, we propose specific measures: investing in public health campaigns, implementing targeted intervention programs, expanding healthcare infrastructure, formulating policies promoting healthy habits, and encouraging research in pediatric metabolic health. By adopting these strategies, China can effectively tackle the growing metabolic health burden among its youth, laying the foundation for a healthier, more productive future population.
