# Supplementary material for: Trends and predictions in demographic structures and metabolic health challenges for Chinese children and adolescents: insights from multiple waves of national school health surveys from 2000 to 2030
Source: Lancet Child Adolesc Health. Author manuscript; Available in PMC 2025 Aug 1. (PMC7617964; doi:10.1016/S2352-4642(25)00140-3)
Supplement: appendix [file EMS207312-supplement-appendix.pdf]

Appendix

Content

1. Anthropometric measurements and statistical analyses 2
2.1Age-specific, sex-specific, and province-specific prevalence prediction 5
2.2 Prevalence and case estimates, and their 95% confidence intervals 7
Table S1. Descriptive characteristics of Chinese children and adolescents aged 7-18 years participating in CNSSCH from 2000 to 2019 10
Table S2. Age - standardized rates (95% confidence intervals) of OWOB, HTN, OWOB coexisting with HTN, severe obesity, severe HTN among Chinese children and adolescents for boys, girls and both sexes, in 2000, 2019, and 2030 11
Table S3. Number of populations (95% confidence intervals, millions) of OWOB, HTN, OWOB coexisting with HTN, severe obesity, severe HTN among Chinese children and adolescents for boys, girls and both sexes, in 2000, 2019, and 2030 12
Table S4. Provincial number of population (95% confidence intervals) of OWOB, HTN, and OWOB coexisting with HTN among Chinese children and adolescents aged 7-18 in 2000, 2019, and 2030, stratified by sex 13
Table S5. Provincial age - standardized rates (95% confidence intervals, %) of OWOB, HTN, and OWOB coexisting with HTN among Chinese children and adolescents aged 7-18 in 2000, 2019, and 2030, stratified by sex 19
Table S6. Chinese demographic trends in 2000, 2019, and 2030 at the national and provincial level 23
Figure S1. The association between the Population Development Index and the birth rate in 2019 24
Figure S2. The association between the Population Development Index and the share of population aged 0-14 in 2019 25
Figure S3. The association between the Population Development Index and the death rate in 2019 26
Figure S4. The association between the Population Development Index and the share of population aged 65+ (%) in 2019 27
Table S8. Predicted estimates of sex-specific and age-specific OWOB prevalence among Chinese children and adolescents aged 7 - 18 years in 2030 28
Figure S5. The association between urbanization rate and the Population Development Index in 2019 29

1. Anthropometric measurements and statistical analyses

1.1 Measures

Anthropometric assessments followed a standardized protocol across all survey sites. Participants were instructed to wear light clothing and stand upright without shoes. Height was measured to the nearest 0.1 cm using a wall-mounted stadiometer, and weight was measured to the nearest 0.1 kg using a scale. Each measurement was taken twice, and the average was recorded. Blood pressure (BP) was measured on the right arm with a mercury sphygmomanometer following standard procedures. Participants rested in a seated position for at least 15 minutes prior to measurement, with the cuff positioned at heart level. The cuff size was chosen so that its bladder covered at least two-thirds of the upper arm circumference. Throughout the procedure, participants remained seated with uncrossed legs, their right arm supported at heart level, and were invited to relax. Systolic BP (SBP) and diastolic BP (DBP) were determined by Korotkoff sounds phases 1 and 5, respectively. Three consecutive measurements were taken, and the mean of these readings was used for analysis.

1.2 Statistical Analysis

Descriptive analysis We calculated the prevalence rates of OWOB, HTN, and OWOB coexisting HTN, stratified by sex, 1-year age group, and province. Subsequently, sex-specific, age-specific, and province-specific prevalence estimates were derived. To adjust for the effect of changes in population structures from 2000 to 2030, age- and sex-specific adjustments were conducted using direct standardization, with children and adolescents aged 7-18 years as the reference standard throughout the period. Standardized coefficients specific to age and sex were based on 2019 national population data for China provided by the UN. To estimate the number of affected individuals in each province, we extrapolated sex-specific, age-specific, and province-specific prevalence rates with corresponding population data for those aged 7-18 for each year, and then summed affected individuals over all sex and age groups in each province. We then aggregated the sums of the province-specific number of affected individuals, and provided the number of affected individuals at the national level. Given limited sample sizes for severe obesity and HTN, national prevalence estimates were computed through sex-stratified and single-year age group stratification. Final case estimates were obtained by applying these stratified prevalence rates to corresponding demographic strata within the national population pyramid.

By subtracting estimated values from 2000 and 2030, changes in the number of affected individuals in each province from 2000 to 2030 were determined. Furthermore, in order to mark the demographic characteristics of provinces at high risk of OWOB, HTN, or OWOB coexisting with HTN, we applied the 2019 data to explore associations with the total fertility rate and aging rate with the age-standardized prevalence rate of OWOB, HTN, or OWOB coexisting with HTN in each province, using scatter plots with fitted linear lines. Correlation coefficients r and corresponding p values were calculated using general linear regression models.

National and provincial projections A new four-category variable was constructed based on the presence or absence of OWOB and HTN: 0 (neither OWOB nor HTN), 1 (isolated OWOB), 2 (isolated HTN), and 3 (OWOB coexisting with HTN). Multinomial (renormalized logistic) regressions were employed to model the prevalence of OWOB, HTN, and OWOB coexisting with HTN as a function of time.[1] Specifically, we modeled the prevalence of each new four-category

variable category i = 1 to 4 at year y as:

$$\text{Prev}(i, y) = \frac{\sigma_i(y)}{\sum_{k=1}^{4} \sigma_k(y)}$$

where

$$\sigma_i(y) = \frac{\exp\left(\beta_0^i + \beta_1^i \cdot y\right)}{1 + \exp\left(\beta_0^i + \beta_1^i \cdot y\right)}$$

This approach ensures that the sum of prevalence across all categories totals 100% each year and enables the assessment of nonlinear trends in prevalence rates. Regressions were conducted nationally across various age and sex groups, as well as provincially across age, sex, and province groups. To account for potential uncertainty, we performed 1000 bootstrap iterations on our datasets to calculate the mean and 95% confidence interval (computed as the 2.5th and 97.5th percentiles of bootstrap values) for all estimates. Specifically, we conducted stratified bootstrap resampling by preserving the stratification structure of the data, which included age, sex, and province. In addition, comparative analysis of standard deviations obtained through maximum likelihood estimation and bootstrap methods revealed a high degree of consistency between the two approaches, as presented in **Table S8**. This convergence suggests that the confidence interval estimates derived from the bootstrap method demonstrate statistical reliability and robustness. The projected prevalence rates (%) of OWOB, HTN, and OWOB with HTN among Chinese boys aged 7-18 years by province and age in 2030 were presented in **Table S5**, **Table S6**, and **Table S7**.

Decomposition methods We conducted decomposition analyses as utilized in the Global Burden of Disease (GBD) framework to decompose the net change in the number of individuals affected by OWOB, HTN, or OWOB coexisting with HTN, at both provincial and national levels, into 3 primary components: population size, age structure, and age-specific prevalence rates.[2,3] This study focused on changes in the number of cases over two time periods: from 2000 to 2019 and from 2000 to 2030. Given the sequence of adding factors may influence the estimates, we estimated the results under all 6 possible sequences of the three factors. The decomposition method estimates the contribution of factors by sequentially introducing each factor into the following equation, where POP_t denotes the child population size in year t, $AgeP_{a,t}$ represents the proportion of the population in age group a in year t, and $Rate_{a,t}$ signifies the prevalence rate among individuals of age a in year t, using the following formula:

$$M_t = \sum_a \left(POP_t \times AgeP_{a,t} \times Rate_{a,t}\right)$$

Statistical analyses were conducted utilizing R statistical software (version 4.1.3; R Foundation for Statistical Computing, Vienna, Austria) in conjunction with Python programming language (version 3.9.7; Python Software Foundation, Wilmington, DE, USA). All statistical tests were two-sided, and $P < 0.05$ was considered statistically significant.

References

1. Ward ZJ, Bleich SN, Cradock AL, et al. Projected US state-level prevalence of adult obesity and severe obesity. *N Engl J Med* 2019; 381: 2440–50.
2. GBD 2016 Causes of Death Collaborators. Global, regional, and national age-sex-specific mortality for 264 causes of death, 1980–2016: a systematic analysis for the Global Burden

of Disease Study 2016. *Lancet* 2017; 390: 1151–210.

3. GBD 2017 Risk Factor Collaborators. Global, regional, and national comparative risk assessment of 84 behavioural, environmental and occupational, and metabolic risks or clusters of risks for 195 countries and territories, 1990–2017: a systematic analysis for the Global Burden of Disease Study 2017. *Lancet* 2018; 392: 1923–94.

2.1Age-specific, sex-specific, and province-specific prevalence prediction

1) Data Subgrouping:

$$\text{Subgroup}_{a,g,p} = \{(y, \text{year}) \mid \text{age} = a, \text{gender} = g, \text{province} = p\}$$

where:

- $a \in$ unique ages
- $g \in \{\text{Male}, \text{Female}\}$
- $p \in$ unique provinces

2) For each subgroup, fit a multinomial regression model:

$$P(y = k \mid \text{year}) = \frac{\exp(\beta_{k0} + \beta_{k1} \cdot \text{year})}{\sum_{j=1}^{4} \exp(\beta_{j0} + \beta_{j1} \cdot \text{year})}$$

where:

y is the four-category variable with values 1, 2, 3, 4 (K=4 is the reference category).

β_{0k} and β_{1k} are model parameters.

$P(y = k)$ is the probability of y taking the k-th category.

3) Set *year* to 2030 and calculate the predicted probabilities for y:

$$P(y = k \mid \text{year} = 2030) = \frac{\exp(\beta_{k0} + \beta_{k1} \cdot 2030)}{\sum_{j=1}^{4} \exp(\beta_{j0} + \beta_{j1} \cdot 2030)}$$

For the reference category $K = 4$:

$$P(y = 4 \mid \text{year} = 2030) = \frac{1}{1 + \sum_{j=1}^{3} \exp(\beta_{0j} + \beta_{1j} \cdot 2030)}$$

4) Bootstrap Resampling

Repeat the following steps 1000 times to estimate the uncertainty of the predictions:

1. **Resample Subgroups**:
   - Randomly sample subgroups (`age`, `gender`, `province`) with replacement.
2. **Refit the Model**:
   - Fit the multinomial regression model on the resampled data.
3. **Predict for 2030**:
   - Predict $P(y = k \mid \text{year} = 2030)$ for each resampled dataset.
4. **Store Predictions**:
   - Save the predicted probabilities for each bootstrap iteration.

For each category k of `y`, calculate the 2.5th and 97.5th percentiles of the predicted probabilities across the 1000 bootstrap iterations:

$$\mathrm{CI}_k = [\mathrm{Quantile}_{0.025}(P_k), \mathrm{Quantile}_{0.975}(P_k)]$$

where P_k is the vector of predicted probabilities for category k across all bootstrap iterations.

2.2 Prevalence and case estimates, and their 95% confidence intervals

1) Variable Definitions:

$P_{a,g,p}$: age-specific, sex-specific, and province-specific prevalence rate.

$P_{g,p}$: sex-specific and province-specific prevalence rate

$P_{a,g}$: age-specific and sex-specific national prevalence rate

P_g: sex-specific national prevalence rate for individuals aged 7 to 18 years

$N_{a,g,p}$: age-specific, sex-specific, and province-specific population count of individuals aged 7 to 18 years

$N_{g,p}$: sex-specific and province-specific population count of individuals aged 7 to 18 years

N_g: sex-specific population count of individuals aged 7 to 18 years at the national level

$C_{g,p}$: sex-specific and province-specific number of cases aged 7 to 18 years

C_g: sex-specific national number of cases aged 7 to 18 years

w_a: age weight, representing the proportion of the population of age a relative to the total population aged 7 to 18 years, $\sum_{a=7}^{18} w_a = 1$

w_p: province weight, representing the proportion of the population of province p aged 7 to 18 years relative to the national population aged 7 to 18 years, $\sum_p w_p = 1$

2) Prevalence, variance and 95% confidence intervals estimations:

Notably, as specified in the Methods section: "Regressions were conducted nationally across various age and sex groups, as well as provincially across age, sex, and province groups."

Sex-specific and provincial-specific prevalence

$$P_{g,p} = \sum_{a=7}^{18} w_a \cdot P_{a,g,p}$$

Variance of $P_{g,p}$

$$\mathrm{Var}(P_{g,p}) = \sum_{a=7}^{18} w_a^2 \cdot \mathrm{Var}(P_{a,g,p})$$

95% confidence intervals for $P_{g,p}$

$$P_{g,p} \pm 1.96 \times \sqrt{\mathrm{Var}(P_{g,p})}$$

Sex-specific prevalence at the national level

$$P_g = \sum_a w_a \cdot P_{a,g}$$

Variance of P_g

$$\text{Var}(P_g) = \sum_a w_a^2 \cdot \text{Var}(P_{a,g})$$

95% confidence intervals for P_g

$$P_g \pm 1.96 \times \sqrt{\text{Var}(P_g)}$$

3) Number of cases, variance and 95% confidence intervals estimations:

Of note, as specified in the Methods section: "To estimate the number of affected individuals in each province, we extrapolated sex-specific, age-specific, and province-specific prevalence rates with corresponding population data for those aged 7-18 for each year, and then summed affected individuals over all sex and age groups in each province. We then aggregated the sums of the province-specific number of affected individuals, and provided the number of affected individuals at the national level. "

Sex-specific and provincial-specific cases

$$C_{g,p} = \sum_a (P_{a,g,p} \times N_{a,g,p})$$
$$= N_{g,p} \times \sum_a (P_{a,g,p} \times w_a)$$

Variance of $C_{g,p}$

$$\text{Var}(C_{g,p}) = \sum_a N_{a,g,p}^2 \cdot \text{Var}(P_{a,g,p})$$
$$= N_{g,p}^2 \cdot \sum_a w_a^2 \cdot \text{Var}(P_{a,g,p})$$

where $N_{g,p} = w_a \cdot N_{a,g,p}$

95% confidence intervals for $C_{g,p}$

$$C_{g,p} \pm 1.96 \times \sqrt{\text{Var}(C_{g,p})}$$

Sex-specific cases at the national level

$$C_g = \sum_p (P_{g,p} \cdot N_{g,p})$$
$$= N_g \cdot \sum_p (P_{g,p} \cdot w_p)$$

where $N_{g,p} = w_p \cdot N_g$

Variance of C_g

$$\mathrm{Var}(C_g) = \sum_p N_{g,p}^2 \cdot \mathrm{Var}(P_{g,p})$$
$$= N_g^2 \cdot \sum_p w_p^2 \cdot \mathrm{Var}(P_{g,p})$$

95% confidence intervals for C_g

$$C_g \pm 1.96 \times \sqrt{\mathrm{Var}(C_g)}$$

Table S1. Descriptive characteristics of Chinese children and adolescents aged 7-18 years participating in CNSSCH from 2000 to 2019

	2000 (N=213931)	2005 (N=236532)	2010 (N=217927)	2014 (N=216764)	2019 (N=221262)	*P* for trend
**Boys**						
Age, years	12.5 (3.5)	12.5 (3.5)	12.5 (3.5)	12.5 (3.4)	12.5 (3.4)	0.72
Height, cm	150.8 (18.3)	152 (18)	153.2 (17.7)	154.4 (17.6)	155.2 (17.9)	0.027
Weight, kg	42.5 (15.6)	43.9 (15.7)	45.5 (16)	47.5 (16.8)	49.2 (18)	0.027
BMI, kg/m2	18 (3.3)	18.3 (3.4)	18.7 (3.5)	19.3 (3.8)	19.7 (4.1)	0.027
SBP, mm Hg	105.3 (11.9)	117.8 (12.2)	106.1 (12.3)	128.2 (12.6)	109.1 (12.6)	0.043
DBP, mm Hg	65.5 (8.5)	77.5 (8.8)	65.5 (8.8)	86.8 (8.8)	67.6 (8.6)	0.13
BMI percentiles (%)	38.8 (29.5)	42.9 (30.8)	47.2 (31.5)	52.6 (31.8)	55.7 (31.7)	0.027
SBP percentiles (%)	44.9 (28.5)	42 (28.5)	45.3 (29.3)	46.2 (29.3)	51.3 (29.6)	0.086
DBP percentiles (%)	59.6 (23.6)	56.2 (24.7)	58.7 (24.6)	59.5 (24)	63.1 (23.5)	0.22
**Girls**						
Age, years	12.5 (3.5)	12.5 (3.5)	12.5 (3.4)	12.5 (3.4)	12.4 (3.4)	0.72
Height, cm	146.6 (14.4)	147.4 (14.1)	148.2 (13.8)	149.3 (13.6)	150 (13.7)	0.027
Weight, kg	39.3 (12.5)	40.2 (12.4)	41.1 (12.4)	42.7 (12.8)	44.1 (13.8)	0.027
BMI, kg/m2	17.8 (3.2)	18 (3.2)	18.2 (3.2)	18.7 (3.4)	19.1 (3.7)	0.027
SBP, mm Hg	102.3 (10.6)	108.8 (10.9)	102.2 (11.1)	123.2 (11.3)	104.8 (11.5)	0.096
DBP, mm Hg	64.7 (8.2)	71 (8.4)	64.4 (8.4)	84.4 (8.3)	66.5 (8.3)	0.27
BMI percentiles (%)	37.5 (26.7)	40.4 (27.4)	43 (28)	47.5 (28.7)	51.4 (28.8)	0.027
SBP percentiles (%)	42.7 (28.2)	38.7 (28)	41.5 (28.9)	42.4 (28.9)	47.2 (29.4)	0.22
DBP percentiles (%)	57.6 (24.1)	53.3 (25)	55.9 (25)	56.7 (24.4)	61.2 (24.1)	0.46

Note: Data are mean (standard deviation). BMI, body mass index; SBP, systolic blood pressure; DBP, diastolic blood pressure.

BMI percentile values was defined using sex- and age-specific 2000 Centers for Disease Control and Prevention guidelines.

Blood pressure percentile values on the basis of age, sex, and height percentiles was defined using the updated BP categories and stages in 2017 American Academy of Pediatrics.

Table S2. Age-standardized rates (95% confidence intervals) of OWOB, HTN, OWOB coexisting with HTN, severe obesity, severe HTN among Chinese children and adolescents for boys, girls and both sexes, in 2000, 2019, and 2030

	2000	2005	2010	2014	2019	2030
Boys						
OWOB	10.4(10.2,10.5)	14.2(14,14.4)	18(17.7,18.2)	23.5(23.2,23.8)	26.7(26.4,27)	41.3(40.6,42.0)
HTN	14.6(14.4,14.8)	13.2(13,13.4)	16.4(16.2,16.6)	16.6(16.4,16.9)	20.2(19.9,20.5)	23.5(22.9,24.1)
OWOB coexisting with HTN	2.6(2.5,2.7)	3.2(3.1,3.3)	4.4(4.3,4.6)	5.7(5.6,5.8)	7.5(7.3,7.6)	12.1(11.8,12.4)
Severe obesity	0.56(0.52,0.61)	0.8(0.75,0.85)	1.15(1.08,1.21)	1.7(1.62,1.78)	2.37(2.28,2.47)	5.44(4.68,6.2)
Severe HTN	1.66(1.58,1.73)	1.54(1.47,1.61)	1.8(1.72,1.88)	2.12(2.04,2.21)	2.68(2.59,2.78)	2.78(2.37,3.19)
Girls						
OWOB	5.6(5.5,5.8)	7.4(7.3,7.6)	9.2(9.1,9.4)	12.9(12.6,13.1)	16(15.8,16.3)	27.2(26.6,27.8)
HTN	10.7(10.6,10.9)	8.7(8.6,8.9)	11.1(10.9,11.3)	10.5(10.3,10.7)	13.7(13.4,13.9)	14.8(14.2,15.4)
OWOB coexisting with HTN	1.2(1.1,1.3)	1.4(1.3,1.5)	1.9(1.8,2)	2.5(2.4,2.6)	3.5(3.4,3.7)	5.8(5.6,6.0)
Severe obesity	0.18(0.15,0.21)	0.23(0.2,0.26)	0.2(0.17,0.22)	0.41(0.37,0.45)	0.7(0.66,0.76)	1.23(0.92,1.54)
Severe HTN	0.78(0.73,0.84)	0.77(0.72,0.82)	0.99(0.94,1.06)	1.02(0.96,1.08)	1.4(1.33,1.47)	1.66(1.47,1.85)
Both sexes						
OWOB	8.1(8,8.2)	11(10.8,11.1)	13.8(13.7,14)	18.4(18.2,18.6)	21.5(21.3,21.7)	34.8(34.3,35.3)
HTN	12.4(12.2,12.5)	10.7(10.6,10.8)	13.5(13.3,13.6)	13.2(13.1,13.4)	16.6(16.4,16.8)	19.5(19.1,19.9)
OWOB coexisting with HTN	1.9(1.8,1.9)	2.3(2.2,2.4)	3.2(3.1,3.3)	4.1(4,4.2)	5.5(5.4,5.6)	9.2(9.0,9.4)
Severe obesity	0.38(0.35,0.41)	0.53(0.5,0.56)	0.69(0.66,0.73)	1.08(1.03,1.12)	1.57(1.52,1.62)	3.5(3.16,3.84)
Severe HTN	1.25(1.21,1.3)	1.19(1.14,1.23)	1.43(1.38,1.48)	1.61(1.56,1.67)	2.09(2.03,2.15)	2.27(2.06,2.48)

Table S3. Number of populations (95% confidence intervals, millions) of OWOB, HTN, OWOB coexisting with HTN, severe obesity, severe HTN among Chinese children and adolescents for boys, girls and both sexes, in 2000, 2019, and 2030

	2000	2019	2030
Boys			
OWOB	14.5 (14.2, 14.9)	28.8 (28.3, 29.3)	39.2(38.5,39.9)
HTN	20.7 (20.3, 21.2)	21.0 (20.6, 21.4)	23.1(22.5,23.7)
OWOB coexisting with HTN	3.8 (3.6, 4.0)	7.8 (7.6, 8.0)	9.5(9.3,9.7)
Severe obesity	0.8 (0.70, 0.9)	2.5 (2.4, 2.6)	4.7(4,5.4)
Severe HTN	2.4 (2.3, 2.56)	2.8 (2.7, 2.9)	3.5(3,4)
Girls			
OWOB	7.1 (6.8, 7.3)	14.6 (14.3, 14.9)	21.4(20.9,21.9)
HTN	14.6 (14.2, 14.9)	11.8 (11.5, 12.1)	11.8(11.3,12.3)
OWOB coexisting with HTN	1.7 (1.5, 1.8)	3.1 (3.0, 3.3)	3(2.9,3.1)
Severe obesity	0.2 (0.2, 0.3)	0.6 (0.6, 0.7)	0.6(0.4,0.8)
Severe HTN	1.1 (1.0, 1.2)	1.2 (1.1, 1.3)	1.3(1.2,1.4)
Both sexes			
OWOB	21.6 (21.1, 22.0)	43.4 (42.8, 44.0)	60.6(59.7,61.5)
HTN	35.3 (34.7, 35.8)	32.8 (32.3, 33.3)	35(34.3,35.7)
OWOB coexisting with HTN	5.4 (5.2, 5.7)	10.9 (10.7, 11.2)	12.5(12.2,12.8)
Severe obesity	1.0 (0.9, 1.1)	3.1 (2.9, 3.2)	5.3(4.8,5.8)
Severe HTN	3.5 (3.3, 3.6)	4.0 (3.9, 4.2)	4.7(4.3,5.1)

Table S4. Provincial number of population (95% confidence intervals) of OWOB, HTN, and OWOB coexisting with HTN among Chinese children and adolescents aged 7-18 in 2000, 2019, and 2030, stratified by sex

Province	OWOB			HTN			OWOB coexisting with HTN		
	2000	2019	2030	2000	2019	2030	2000	2019	2030
**Boys**									
Beijing	17.1(15.9,18.5)	33.4(31.5,35.4)	39.9(36,46)	12.4(11.3,13.5)	18(16.4,19.6)	21.3(18.5,24.1)	4(3.4,4.7)	8.5(7.5,9.7)	11.5(9.7,13.3)
Tianjin	9.7(8.6,11.1)	29.4(28,31)	40.9(37.6,44.2)	15.6(14.1,17.2)	18.9(17.8,20.2)	22.3(20.1,24.5)	2(1.5,2.6)	10.2(9.4,11.2)	16.4(14.5,18.3)
Hebei	159.5(147.5,172.1)	224.3(211.5,237.7)	247.7(243.5,289.2)	202.2(188.8,216.3)	184.4(173.1,196.3)	185(161,209)	54.8(47.9,62.5)	68.5(61.5,76)	78.1(68.1,88.1)
Shanxi	48.4(43.8,53.4)	65.4(61.2,69.9)	80.9(76.3,95.9)	49.2(44.7,54.2)	52.4(48.7,56.4)	55.5(45.2,65.8)	12.5(10.3,15.2)	18.7(16.5,21.2)	19.6(16,23.2)
Inner Mongolia	24(21.3,27)	46.7(44.1,49.5)	71.6(67.6,79.4)	53.8(50,58)	37.8(35.4,40.2)	39.8(35,44.6)	7.9(6.5,9.8)	16.3(14.8,18)	22.5(19.4,25.6)
Liaoning	53.4(50,57.1)	74(69.9,78.2)	97.3(91.8,105.5)	84.3(80,88.7)	76.2(72.1,80.5)	78.9(72.3,85.5)	17.5(15.5,19.6)	36.4(33.5,39.4)	51.9(47,56.8)
Jilin	20.2(17.8,22.7)	43.3(40.5,46.2)	63.7(60,71.6)	41.2(38,44.7)	38.5(35.8,41.4)	40.8(34.5,47.1)	5.3(4.1,6.7)	13.5(12,15.3)	18.9(16,21.8)
Heilongjiang	46.2(42.1,50.6)	67.7(64.1,71.4)	85.2(78.9,93.5)	76.6(71.4,82)	38.7(36,41.6)	45.9(40.9,50.9)	11.8(9.8,14.1)	19.9(18,21.9)	30.2(26.2,34.2)
Shanghai	15.4(14.5,16.3)	34.8(32.8,36.9)	47.5(43.1,52.3)	13.4(12.6,14.3)	24.9(23.3,26.7)	26.4(23,29.8)	3.3(2.9,3.7)	11.3(10.2,12.5)	16.1(13.7,18.5)
Jiangsu	102.1(93.5,111.4)	117.2(109.3,125.6)	115.8(100,141)	54.7(48.6,61.4)	95.1(88.3,102.3)	118.9(97.6,140.2)	11.5(8.8,14.8)	30.1(26.3,34.4)	28.7(21.4,36)
Zhejiang	50.8(46,55.9)	79.6(74.2,85.3)	94.6(86,111.5)	22.5(19.5,25.8)	55.8(51.6,60.4)	90.8(74.4,107.2)	3.9(2.7,5.5)	17.6(15.1,20.3)	18.9(13.7,24.1)
Anhui	56.1(49.5,63.4)	163.5(154.3,173.3)	262.8(237.4,278.5)	112.5(103.3,122.4)	163.4(154.2,173)	206.8(183.7,229.9)	14.3(11.1,18.2)	60.6(55,66.6)	114.7(100.2,129.2)
Fujian	58.9(53.9,64.2)	89.5(84.3,95)	102.1(89.4,119.1)	38.5(34.5,42.7)	39.2(35.7,42.9)	38.9(29.3,48.5)	9.3(7.4,11.6)	17.7(15.4,20.3)	15.2(10.2,20.2)

Jiangxi	32.6(28.4,37.5)	98.6(91.8,105.9)	158.5(146,184.3)		43.9(39.1,49.2)	79.5(73.4,86.2)	101.3(78.3,124.3)		6.4(4.6,8.7)	25.4(22,29.2)	31.1(21.9,40.3)
Shandong	203.2(190.1,217.2)	367(349.9,384.8)	424.5(408.5,471)		258.7(244.1,274)	250.4(236.7,264.8)	256.5(233,280)		88.6(80.1,97.9)	122.6(113,132.9)	145.4(129.9,160.9)
Henan	119.5(107.3,132.7)	265.5(248.2,283.6)	336.8(318.1,400.6)		236.7(219.6,254.7)	150(137.2,163.6)	128.9(104.6,153.2)		33.9(27.6,41.3)	63.9(55.6,73)	71.4(58.1,84.7)
Hubei	31.2(26.6,36.4)	114.3(107.1,121.9)	198.8(187,222.5)		93.2(85.2,101.8)	116.1(108.7,123.8)	133.5(114.9,152.1)		6.6(4.5,9.2)	40.3(36,44.9)	65.3(53.5,77.1)
Hunan	62.7(55.9,70.1)	168.7(158.2,179.7)	240.4(220.9,279)		57.4(51.1,64.4)	112.1(103.7,121)	106.1(79.6,132.6)		10.4(7.7,13.6)	34.5(29.9,39.7)	29(20,38)
Guangdong	55.5(46.1,66.4)	126.1(115.9,137)	182.2(165.4,231.6)		85.3(73.9,98)	97.4(88.5,107)	119.6(69.5,169.7)		6.8(3.8,11.3)	24.1(19.7,29.1)	17.3(5.1,29.5)
Guangxi	52(46.4,58.1)	102.5(95.1,110.4)	157.9(137.6,184.4)		43.9(38.9,49.3)	51.1(45.9,56.7)	57.5(40.9,74.1)		9.2(6.9,11.9)	16.4(13.5,19.7)	20(13.8,26.2)
Hainan	6.9(6.1,7.9)	18.3(17,19.6)	26.4(23.5,30.6)		14(12.7,15.3)	8.8(7.9,9.7)	11(8.3,13.7)		1.4(1,1.8)	3.2(2.6,3.7)	3.1(2.4,3.8)
Chongqing	18.6(16,21.4)	52.1(48.3,56.2)	90.5(79.5,107)		31.9(28.6,35.6)	23.4(20.9,26.1)	20.4(11.5,29.3)		3.7(2.6,5.1)	5.7(4.5,7.1)	4(2.1,5.9)
Sichuan	59.5(52,67.9)	112.6(104.1,121.5)	155.4(138.8,197.8)		126.7(115.8,138.4)	78.1(71.1,85.6)	90.1(58.5,121.7)		16.4(12.5,21)	22.3(18.6,26.5)	14.1(6,22.2)
Guizhou	18.9(15.7,22.6)	70.4(65.1,76)	116.3(104.5,144.3)		41.7(37,46.8)	53.4(49,58.2)	65.4(45.6,85.2)		3.7(2.4,5.5)	13.6(11.4,16.1)	12.2(4.3,20.1)
Yunnan	44(37.9,50.8)	82.9(77.3,88.8)	121.4(112.7,146.6)		106.9(97.5,116.9)	64.9(60.1,70.1)	58.9(43.3,74.5)		14.8(11.3,19)	17.5(15.1,20.3)	15(10.2,19.8)
Tibet	/	4.3(4,4.7)	4.7(3.9,6.4)		/	9.6(9.1,10.2)	10.2(6.5,13.9)		/	1.4(1.2,1.7)	1.5(1.1,1.9)
Shaanxi	34.6(30.6,39)	71(66.8,75.4)	116.5(106.8,132.4)		64.4(59,70.1)	35.8(32.8,38.9)	33.5(27.1,39.9)		8.8(6.9,11.2)	13.5(11.7,15.4)	17(12.6,21.4)
Gansu	22.6(19.9,25.7)	49.7(46.3,53.3)	69.7(67.7,84.2)		25.9(23.1,29)	41.1(38.1,44.3)	53.2(42.7,63.7)		3.3(2.4,4.7)	12.2(10.6,14.1)	12.6(9.4,15.8)
Qinghai	2.6(1.9,3.4)	11.7(10.8,12.7)	15.9(14.8,20.7)		11.7(10.1,13.4)	15.2(14.1,16.3)	11.1(8.5,13.7)		0.9(0.5,1.5)	4.9(4.3,5.5)	3.7(2.2,5.2)
Ningxia	3.4(2.8,4)	15.9(14.7,17.1)	26.3(24.2,30)		18.1(16.9,19.4)	16.1(14.9,17.4)	18.4(15.5,21.3)		1.8(1.4,2.3)	4.8(4.2,5.6)	8(6.5,9.5)

Xinjiang	21.5(17.5,26.2)	82(77,87.2)	130(118.3,149.8)		32.7(27.8,38.3)	54(50.1,58.2)	68(57.6,78.4)		3.9(2.3,6.3)	23.4(20.8,26.2)	36(28.6,43.4)
**Girls**											
Beijing	9.2(8.3,10.1)	16(14.8,17.4)	20.4(17.3,24.3)		7(6.2,7.8)	7(6.1,8)	6.8(5.1,8.5)		1.8(1.4,2.3)	3.2(2.6,3.9)	2.8(2.2,3.4)
Tianjin	6.3(5.4,7.3)	17.2(16.2,18.3)	25.2(23.1,28.1)		10.8(9.6,12.1)	10.6(9.8,11.5)	9.3(7.7,10.9)		1.4(1,1.9)	4.9(4.4,5.5)	5.1(4.1,6.1)
Hebei	89(80.6,98)	120.5(112,129.5)	131.9(125.3,156)		138.7(128.2,150)	103.9(96.1,112.2)	99.9(79.8,120)		26.5(22,31.7)	32.4(28,37.2)	32.2(27.1,37.3)
Shanxi	23.6(20.5,27)	34.9(32,37.9)	45.5(39.6,54.2)		39.8(35.9,44.1)	28.3(25.7,31)	24.6(17.2,32)		4.4(3.2,6.1)	7.6(6.3,9.1)	5.9(3.9,7.9)
Inner Mongolia	12.9(11.1,14.9)	24.2(22.4,26)	40.4(37.1,46.3)		32.6(29.8,35.7)	20.4(18.8,22.1)	24(18.8,29.2)		3.4(2.5,4.5)	6.2(5.3,7.2)	8(6.4,9.6)
Liaoning	28.9(26.5,31.5)	37.3(34.6,40.2)	50.1(43.6,53.9)		59.8(56.3,63.5)	52.7(49.4,56)	47.2(41.7,52.7)		8.5(7.2,10)	16.2(14.4,18.1)	16.2(13.4,19)
Jilin	14.7(12.9,16.8)	24.7(22.8,26.9)	37.6(34.1,44.1)		35.9(33,39)	23.6(21.5,25.9)	15.5(11.2,19.8)		3.5(2.6,4.6)	6.4(5.4,7.6)	5.2(3.7,6.7)
Heilongjiang	22.4(19.7,25.4)	35.4(33.1,37.9)	50.8(45,56.9)		46.7(42.8,50.8)	21.3(19.4,23.3)	17.6(14.1,21.1)		4.5(3.3,5.9)	7.6(6.5,8.9)	7.6(5.9,9.3)
Shanghai	7.7(7.1,8.3)	16.6(15.3,18)	21.5(19,25.4)		7.2(6.7,7.9)	11.8(10.7,13)	11.4(8.8,14)		1.1(0.9,1.3)	3.8(3.2,4.5)	3.5(2.3,4.7)
Jiangsu	44(38.6,49.9)	53.4(48.5,58.6)	58.7(50.4,74.7)		27.8(23.6,32.5)	47.7(43.2,52.5)	51(30.5,71.5)		2.6(1.4,4.3)	9.2(7.3,11.5)	7.7(4.1,11.3)
Zhejiang	25.8(22.6,29.3)	33.7(30.5,37.3)	36.6(31.8,46.6)		10.4(8.4,12.7)	31.7(28.7,35)	63(38.3,87.7)		1.7(0.9,2.8)	5.6(4.3,7.1)	4.9(2.5,7.3)
Anhui	27.2(22.9,32.2)	78.6(72.6,85)	121.7(100.8,137.4)		60.2(53.7,67.3)	102.2(95.3,109.5)	117.4(91,143.8)		5.2(3.4,7.7)	21.8(18.7,25.3)	24.4(15,33.8)
Fujian	21.1(18.3,24.3)	39(35.8,42.4)	41.4(33.2,56.3)		19.3(16.6,22.3)	17.2(15.1,19.5)	16.1(3.4,28.8)		2.2(1.4,3.4)	5.1(4,6.4)	2.8(-2.1,7.7)
Jiangxi	10.7(8.5,13.4)	43.8(39.6,48.4)	79.2(64.3,95.8)		29.9(26.1,34.2)	39.1(35.1,43.5)	47.1(25,69.2)		1.2(0.6,2.4)	8.6(6.8,10.8)	10.7(2.2,19.2)
Shandong	95.5(87,104.8)	198.5(186.8,210.8)	257.5(252.9,301.5)		177.2(165.4,189.6)	153.7(143.7,164.2)	155.6(128.4,182.8)		35.3(30.1,41.1)	54.4(48.5,60.9)	48.7(38.4,59)
Henan	63.8(55.3,73.2)	135(123.6,147.3)	203.7(180.4,247)		186.6(172,202.2)	68.3(60.3,77.1)	46.6(35.5,57.7)		22.8(17.8,28.8)	21.7(17.3,26.9)	18.3(14.2,22.4)
Hubei	12.3(9.6,15.6)	55.2(50.8,60)	99.6(87.4,118.5)		49(43.4,55.1)	65.7(60.7,71)	73.1(50.1,96.1)		1.6(0.7,3.1)	14.8(12.6,17.4)	21.5(12.1,30.9)
Hunan	24.8(20.8,29.3)	86.1(79.3,93.4)	146.3(129.4,178.2)		41.9(36.7,47.7)	71.4(65.2,78)	69.3(41.8,96.8)		2.4(1.3,4.1)	17.4(14.5,20.9)	19.4(6,32.8)
Guangdong	34.1(27.2,42.3)	56.2(50,63)	66.3(49.8,92.1)		49.9(41.5,59.6)	53.4(47.4,60)	51.8(4.7,98.9)		6(3.3,10.1)	10.2(7.7,13.4)	5.8(-1,12.6)
Guangxi	24.1(20.5,28.2)	46.6(42,51.6)	77.4(60.5,93.5)		41.5(36.7,46.8)	27.1(23.7,30.9)	33.3(17.1,49.5)		3.9(2.5,5.8)	4(2.8,5.6)	5.1(2.9,7.3)

Hainan	3.6(3,4.3)	9.7(8.9,10.6)	13.4(9.6,16)		8(7.1,9.1)	6.1(5.5,6.9)	11.9(5.6,18.2)		0.8(0.5,1.2)	1(0.8,1.4)	0.8(0.3,1.3)
Chongqing	7.5(6,9.3)	27.9(25.1,30.9)	51.3(38,65.8)		24.8(22,28)	12.3(10.5,14.4)	14.8(3.3,26.3)		1.5(0.9,2.4)	1.5(0.9,2.4)	1.1(0.5,1.7)
Sichuan	28.5(23.7,34.1)	60.1(54.5,66.2)	84.2(66.6,111.1)		116.8(106.8,127.7)	39.3(34.7,44.3)	45.4(18.6,72.2)		6.6(4.3,9.6)	10.7(8.4,13.5)	8.9(1.8,16)
Guizhou	10.4(8.2,13)	43(39.3,47)	79.6(71.5,102)		36.1(31.8,40.8)	34.3(31,37.9)	25.6(8.8,42.4)		1.8(1,3.2)	7.8(6.3,9.6)	7.7(2.4,13)
Yunnan	22.5(18.5,27.2)	36.6(33.2,40.3)	55(43.4,68.7)		90.7(82.4,99.7)	35.3(32,38.9)	22.9(10.6,35.2)		6.7(4.6,9.5)	5.9(4.6,7.4)	4.8(1.8,7.8)
Tibet	/	3.4(3.1,3.7)	4.7(3.3,6)		/	7.8(7.3,8.3)	6.8(4.4,9.2)		/	1(0.8,1.2)	1(0.6,1.4)
Shaanxi	13.6(11.2,16.3)	36.7(33.9,39.6)	76.9(67.4,91)		49.6(45.1,54.5)	18.6(16.6,20.7)	10.6(6.7,14.5)		3.1(2,4.5)	4.8(3.9,6)	4.1(2.1,6.1)
Gansu	11.1(9.3,13.2)	30.5(28.1,33.1)	51.7(47.5,63.3)		13.9(11.9,16.2)	24.3(22.2,26.6)	26.8(18.3,35.3)		1.6(1,2.6)	6(5,7.2)	3.4(1.1,5.7)
Qinghai	1.1(0.7,1.6)	6.2(5.6,6.9)	11.9(10,15.1)		8.2(7,9.6)	11(10.2,11.9)	8.4(5.5,11.3)		0.3(0.1,0.7)	2.1(1.7,2.5)	1.6(1,2.2)
Ningxia	2.1(1.7,2.5)	7.8(7,8.7)	12.3(10.2,14.8)		14.5(13.5,15.6)	9.6(8.7,10.6)	7.3(4.7,9.9)		0.7(0.5,1)	1.9(1.5,2.4)	1.4(0.6,2.2)
Xinjiang	6.9(4.8,9.6)	40.3(37.1,43.7)	83(68.4,99.3)		20.7(17,25.1)	23.1(20.8,25.7)	20.8(14.6,27)		2.3(1.2,4.2)	7.8(6.5,9.4)	9(5.6,12.4)
**Both sexes**											
Beijing	13.2(11,15.8)	49.4(47.1,51.8)	62(55.4,68.5)		19.5(18.1,20.9)	24.9(23.1,26.9)	25.5(22.8,28.2)		5.9(5.1,6.7)	11.7(10.5,13)	13.2(11.6,14.8)
Tianjin	24.9(21.7,28.6)	46.7(44.9,48.5)	66.7(62.4,71.1)		26.2(24.3,28.2)	29.6(28.1,31.1)	29.1(26.8,31.4)		3.4(2.7,4.1)	15.2(14.2,16.3)	19.9(18,21.8)
Hebei	143.3(123.1,166.8)	344.8(329.4,360.8)	408.3(378.5,438.4)		342.1(324.9,360.1)	288.4(274.6,302.7)	270.1(242.5,297.7)		81.9(73.5,91)	100.8(92.6,109.6)	111.6(99.9,123.3)
Shanxi	42.3(34.5,53.7)	100.3(95.1,105.6)	133.5(120,146.6)		89.3(83.2,95.8)	80.7(76.1,85.5)	72.8(61.7,83.9)		17.1(14.5,20.1)	26.3(23.7,29.1)	24(20,28)
Inner Mongolia	52.4(42.9,64.4)	70.9(67.7,74.2)	115.4(107.4,123.5)		87.4(82.5,92.5)	58.2(55.3,61.2)	58.7(52.3,65.1)		11.3(9.5,13.3)	22.5(20.7,24.4)	28.9(25.6,32.2)
Liaoning	133(112.4,157.5)	111.3(106.4,116.4)	147.8(139.3,156.5)		143.9(138.4,149.7)	128.9(123.6,134.3)	130.2(120.6,139.8)		26(23.6,28.5)	52.5(49.2,56.1)	65.9(60.5,71.3)
Jilin	42.1(33.8,52.1)	68(64.6,71.6)	105.1(96.7,113.3)		77.7(73.4,82.3)	62.1(58.7,65.7)	51(44.6,57.4)		8.7(7.2,10.3)	19.9(18.1,22)	21.7(19.1,24.3)
Heilongjiang	74.1(63.8,85.7)	103.1(98.8,107.5)	137.5(127.6,147.5)		124.8(118.3,131.7)	60(56.7,63.4)	56.9(51.8,62)		16.5(14.1,19.2)	27.5(25.3,29.8)	33.5(30.1,36.9)
Shanghai	17.7(14,23.2)	51.4(49,53.9)	70.2(64.1,76.3)		21.1(20,22.2)	36.8(34.8,38.9)	35(31.3,38.7)		4.4(3.9,4.9)	15.1(13.8,16.5)	17.6(15.4,19.8)

Jiangsu	51.8(38.5,72.5)	170.6(161.3,180.4)	183.3(156,210.2)		84.7(77,92.9)	142.8(134.6,151.4)	153(126.2,179.8)		14.7(11.6,18.4)	39.4(35,44.1)	33.1(25.8,40.4)
Zhejiang	29.7(21.9,40.6)	113.4(107,120)	138.8(122.7,154.5)		33.7(30,37.8)	87.5(82.3,93)	138.2(112.4,164)		5.6(4.2,7.5)	23.1(20.4,26.2)	22.6(17.7,27.5)
Anhui	194.5(155.5,244)	242.1(231,253.6)	378.5(350.1,406.9)		175.6(164.1,187.8)	265.6(254,277.6)	322.3(284,360.6)		19.9(16.1,24.3)	82.4(76,89.3)	127.1(112.1,142.1)
Fujian	24.4(16.2,49.4)	128.6(122.4,135)	149.7(129.9,169.6)		58.8(53.9,64)	56.4(52.3,60.7)	50.8(36.2,65.4)		11.6(9.5,14.1)	22.8(20.2,25.6)	17.2(10.4,24)
Jiangxi	49.8(35.2,85.6)	142.4(134.4,150.9)	246(219.5,272.3)		74.3(68.1,81.1)	118.6(111.2,126.5)	132.4(105,159.8)		7.7(5.7,10.2)	34(30.1,38.3)	37.1(26.3,47.9)
Shandong	224.9(198.8,254.9)	565.5(544.8,587)	718(675,762.1)		438.2(419.3,457.8)	404.1(387.1,421.8)	363.3(333.3,393.3)		125.2(115,136)	177(165.7,189)	176.6(160.8,192.4)
Henan	124(101,152)	400.5(379.7,422.2)	574.9(516.2,631.9)		422.2(399.7,445.7)	218.2(203.1,234.3)	168.8(145.9,191.7)		56.2(48.1,65.3)	85.6(76.1,95.9)	88.2(75.9,100.5)
Hubei	142.5(109.8,184.7)	169.5(161,178.4)	308.5(283.9,332.4)		144.5(134.5,155)	181.7(172.8,191)	188(161.2,214.8)		8.1(5.9,11)	55.1(50.2,60.3)	80.2(66.8,93.6)
Hunan	53.7(38.2,86.4)	254.8(242.3,267.9)	404.9(363.4,447)		101.2(92.8,110.2)	183.5(173.1,194.5)	158.5(125,192)		12.9(10,16.5)	52(46.4,58)	43.9(29.5,58.3)
Guangdong	32.1(20.5,61.8)	182.4(170.3,195)	270.5(230.1,310.3)		136.6(122.2,152.4)	150.8(140,162.2)	160.5(97.2,223.8)		12.3(8.2,17.8)	34.3(29.2,40.1)	23.7(10.2,37.2)
Guangxi	29.5(21.7,41.3)	149.1(140.4,158.3)	238.7(209.7,268.5)		84.9(78,92.3)	78.2(71.9,84.9)	83.3(63.1,103.5)		13(10.4,16.2)	20.4(17.2,24)	23.1(17.4,28.8)
Hainan	5.5(4.3,7.2)	28(26.5,29.6)	40(35.4,44.6)		22.1(20.4,23.8)	14.9(13.8,16.1)	23.2(16.1,30.3)		2.1(1.7,2.7)	4.2(3.6,4.9)	4.1(3.2,5)
Chongqing	7.5(5.2,11.4)	80(75.2,85)	145.5(123.6,167.1)		56.6(52.2,61.3)	35.7(32.6,39)	31.5(19.7,43.3)		5.2(3.9,6.8)	7.2(5.8,8.9)	4.9(3.2,6.6)
Sichuan	35.3(23.8,99)	172.7(162.5,183.4)	257.8(217.9,298.1)		242.8(228,258.3)	117.4(108.9,126.3)	118.3(86.2,150.4)		23.5(18.8,28.9)	33(28.6,37.9)	21.6(13.1,30.1)
Guizhou	29.6(17.8,83.4)	113.4(106.9,120.2)	211.3(184.2,238.2)		79(72.5,85.9)	87.7(82.2,93.7)	79.8(59,100.6)		5.5(3.9,7.7)	21.4(18.7,24.5)	18.6(10.9,26.3)

Yunnan	28.4(20.7,41.9)	119.5(112.9,126.4)	186.5(163.9,209.2)		199(186.3,212.3)	100.2(94.3,106.4)	72.7(55.8,89.6)		21.3(17.2,26)	23.4(20.6,26.6)	18.7(13.9,23.5)
Tibet	/	7.7(7.2,8.2)	9.8(8,11.5)		/	17.4(16.7,18.2)	17.4(13,21.8)		/	2.4(2.2,2.7)	2.8(2.2,3.4)
Shaanxi	34.5(24.9,47.4)	107.7(102.6,112.9)	199.1(180.7,217.8)		114.8(107.6,122.3)	54.4(50.8,58.1)	39(33.1,44.9)		11.8(9.5,14.5)	18.3(16.2,20.5)	18.2(14.7,21.7)
Gansu	27.3(19.7,40.7)	80.2(76,84.5)	131.4(118.7,144.1)		40(36.5,43.8)	65.4(61.7,69.3)	71.3(60.3,82.3)		4.9(3.8,6.4)	18.3(16.3,20.4)	15.1(11.6,18.6)
Qinghai	7.6(5.4,10.9)	17.9(16.8,19.1)	30.4(26.6,34.2)		19.8(17.9,22)	26.2(24.9,27.7)	19.6(15.5,23.7)		1.2(0.8,1.8)	6.9(6.3,7.7)	5.7(3.9,7.5)
Ningxia	11.2(8.1,16.1)	23.7(22.3,25.2)	39.7(35.9,43.5)		32.3(30.7,34)	25.7(24.2,27.3)	22.7(19.6,25.8)		2.5(2.1,3)	6.7(6,7.6)	8.6(7.3,9.9)
Xinjiang	38.5(28.9,51.3)	122.2(116.3,128.4)	218.3(193.3,243.6)		54(47.7,60.9)	77.2(72.6,82)	77.1(67.8,86.4)		6.1(4.1,8.8)	31.2(28.3,34.4)	38.6(32.7,44.5)

Note: The estimates for Tibet from 2000 to 2030 have been replaced by the numerical estimates from 2019 to 2030.

Table S5. Provincial age-standardized rates (95% confidence intervals, %) of OWOB, HTN, and OWOB coexisting with HTN among Chinese children and adolescents aged 7-18 in 2000, 2019, and 2030, stratified by sex

	OWOB			HTN			OWOB coexisting with HTN		
	2000	2019	2030	2000	2019	2030	2000	2019	2030
**Boys**									
Beijing	18.3(16.9,19.7)	32.7(30.9,34.7)	42.8(37.6,48)	13.4(12.2,14.6)	17.6(16.1,19.2)	19.7(17.1,22.3)	4.3(3.7,5.1)	8.4(7.4,9.5)	10.8(9.1,12.5)
Tianjin	11.6(10.2,13.1)	40.3(38.3,42.4)	59.7(54.9,64.5)	18.5(16.7,20.4)	25.9(24.3,27.6)	29.4(26.5,32.3)	2.4(1.8,3.1)	14(12.9,15.3)	21.8(19.3,24.3)
Hebei	20.6(19.1,22.3)	34.3(32.4,36.4)	43.4(39.7,47.1)	26.4(24.6,28.2)	28.2(26.5,30)	27.8(24.2,31.4)	7.1(6.2,8.1)	10.5(9.4,11.6)	12.6(11,14.2)
Shanxi	11.4(10.3,12.5)	25(23.4,26.7)	35(31,39)	11.7(10.6,12.8)	20(18.6,21.5)	20(16.3,23.7)	2.9(2.4,3.6)	7.1(6.3,8.1)	7.4(6,8.8)
Inner Mongolia	9.4(8.3,10.5)	30.9(29.2,32.8)	51.8(47.6,56)	21.6(20.1,23.3)	25(23.4,26.6)	25.5(22.4,28.6)	3.1(2.5,3.8)	10.8(9.8,11.9)	14.8(12.8,16.8)
Liaoning	14.1(13.2,15.1)	36.5(34.5,38.7)	51.9(48.3,55.5)	22.6(21.5,23.8)	37.7(35.6,39.8)	40.5(37.1,43.9)	4.6(4.1,5.2)	18(16.6,19.5)	25.9(23.4,28.4)
Jilin	7.9(7,8.9)	29.9(28,31.9)	48.4(44.1,52.7)	16.2(15,17.6)	26.6(24.7,28.6)	26.4(22.3,30.5)	2(1.6,2.5)	9.3(8.3,10.5)	12.2(10.3,14.1)
Heilongjiang	13.2(12.1,14.5)	39.6(37.5,41.8)	53.7(49.1,58.3)	22.3(20.8,23.9)	22.6(21.1,24.3)	24.9(22.2,27.6)	3.4(2.8,4.1)	11.6(10.5,12.8)	16.3(14.2,18.4)
Shanghai	15.1(14.2,16)	31.4(29.6,33.3)	45.9(41.5,50.3)	13.4(12.6,14.3)	22.5(21,24.1)	23.1(20.1,26.1)	3.2(2.8,3.6)	10.2(9.2,11.3)	13.7(11.6,15.8)
Jiangsu	13.9(12.8,15.2)	23.1(21.5,24.8)	25.3(21,29.6)	7.7(6.8,8.6)	18.7(17.4,20.2)	21.8(17.9,25.7)	1.6(1.2,2.1)	5.9(5.2,6.8)	5.4(4,6.8)
Zhejiang	12(10.9,13.2)	22.9(21.3,24.5)	30.2(26.3,34.1)	5.5(4.8,6.3)	16(14.8,17.3)	24.4(20,28.8)	0.9(0.6,1.3)	5(4.3,5.8)	5.3(3.9,6.7)
Anhui	7.4(6.5,8.3)	30.1(28.4,31.9)	50.6(46.6,54.6)	14.9(13.7,16.3)	30.1(28.4,31.9)	39.4(35,43.8)	1.9(1.4,2.4)	11.2(10.1,12.3)	20.2(17.7,22.7)
Fujian	14.7(13.4,16)	29.3(27.6,31.1)	36.3(31.1,41.5)	9.8(8.8,10.9)	12.8(11.7,14)	12.1(9.1,15.1)	2.3(1.8,2.9)	5.8(5.1,6.6)	4.9(3.3,6.5)
Jiangxi	6.1(5.3,7)	22.9(21.3,24.6)	40.8(36.1,45.5)	8.4(7.5,9.4)	18.5(17,20)	21.6(16.7,26.5)	1.2(0.9,1.6)	5.9(5.1,6.8)	6.6(4.6,8.6)
Shandong	21.2(19.8,22.6)	45.2(43,47.3)	57.6(53.5,61.7)	27.4(25.8,29)	30.8(29.1,32.6)	29.5(26.8,32.2)	9.3(8.4,10.3)	15.1(13.9,16.3)	17.1(15.3,18.9)
Henan	9.9(8.9,11)	28.6(26.7,30.5)	41.2(36.5,45.9)	19.8(18.3,21.3)	16.1(14.8,17.6)	13.8(11.2,16.4)	2.8(2.3,3.4)	6.9(6,7.9)	7.9(6.4,9.4)
Hubei	4.6(3.9,5.3)	27.1(25.4,28.9)	51.7(47.2,56.2)	13.7(12.5,14.9)	27.5(25.8,29.4)	30.1(25.9,34.3)	0.9(0.6,1.3)	9.5(8.5,10.7)	15(12.3,17.7)
Hunan	8.7(7.8,9.7)	26.7(25,28.4)	42.1(37.2,47)	8.2(7.3,9.2)	17.7(16.4,19.1)	15.6(11.7,19.5)	1.5(1.1,1.9)	5.5(4.7,6.3)	4.4(3,5.8)
Guangdong	5.2(4.3,6.2)	15(13.8,16.3)	25.2(21,29.4)	8.3(7.2,9.5)	11.6(10.6,12.8)	13.6(7.9,19.3)	0.6(0.4,1)	2.9(2.4,3.5)	2.2(0.6,3.8)
Guangxi	8.8(7.9,9.9)	20.7(19.2,22.3)	34.6(29.6,39.6)	7.6(6.7,8.5)	10.3(9.3,11.4)	11.1(7.9,14.3)	1.6(1.2,2)	3.3(2.7,4)	3.9(2.7,5.1)
Hainan	6.4(5.6,7.4)	21.7(20.2,23.3)	34.2(29.7,38.7)	13.3(12.1,14.6)	10.5(9.4,11.6)	14.1(10.7,17.5)	1.3(0.9,1.7)	3.7(3.1,4.4)	4(3.1,4.9)
Chongqing	5.4(4.7,6.3)	21.8(20.2,23.5)	41.5(35.4,47.6)	9.4(8.4,10.5)	9.8(8.7,10.9)	8(4.5,11.5)	1.1(0.8,1.5)	2.4(1.9,3)	1.7(0.9,2.5)
Sichuan	6.3(5.5,7.2)	17.9(16.6,19.3)	28.5(23.5,33.5)	13.4(12.3,14.6)	12.4(11.3,13.6)	13.1(8.5,17.7)	1.7(1.3,2.2)	3.5(3,4.2)	2.2(0.9,3.5)
Guizhou	3.4(2.9,4.1)	19.1(17.6,20.6)	35.9(30.2,41.6)	7.9(7.1,8.9)	14.5(13.3,15.8)	16.2(11.3,21.1)	0.7(0.4,1)	3.7(3.1,4.4)	3.2(1.1,5.3)
Yunnan	7.7(6.7,8.9)	20.7(19.3,22.1)	34.4(29.9,38.9)	19.2(17.6,21)	16.2(15,17.5)	13.6(10,17.2)	2.5(2,3.3)	4.4(3.8,5.1)	3.7(2.5,4.9)

Tibet	/	10.3(9.5,11.3)	13(9.9,16.1)		/	23(21.7,24.3)	26.4(16.7,36.1)		/	3.4(2.9,4)	4.5(3.4,5.6)
Shaanxi	7.6(6.7,8.6)	27.6(25.9,29.3)	49.4(44.1,54.7)		14.5(13.3,15.8)	13.9(12.7,15.1)	12(9.7,14.3)		1.9(1.5,2.4)	5.2(4.5,6)	6(4.4,7.6)
Gansu	6.5(5.7,7.4)	24(22.3,25.7)	39(34.7,43.3)		7.6(6.7,8.5)	19.8(18.4,21.4)	23.8(19.1,28.5)		1(0.7,1.3)	5.9(5.1,6.8)	6(4.5,7.5)
Qinghai	3.9(2.9,5.2)	21.5(19.9,23.3)	34.8(29,40.6)		17.7(15.4,20.3)	28(26,30.1)	21.2(16.2,26.2)		1.4(0.8,2.2)	9(7.9,10.2)	7.5(4.4,10.6)
Ningxia	4.2(3.5,4.9)	24.2(22.4,26.1)	43.9(39.2,48.6)		22.7(21.2,24.3)	24.4(22.6,26.4)	25.7(21.7,29.7)		2.2(1.8,2.8)	7.4(6.4,8.5)	11.5(9.3,13.7)
Xinjiang	8.4(6.9,10.3)	29.4(27.6,31.3)	51.2(45.2,57.2)		13(11,15.2)	19.4(18,20.9)	22.2(18.8,25.6)		1.5(0.9,2.3)	8.4(7.5,9.4)	11.7(9.3,14.1)
**Girls**											
Beijing	10.7(9.6,11.8)	18.4(16.9,19.9)	24.8(20.7,28.9)		8(7.1,8.9)	8(7,9.2)	7.8(5.8,9.8)		2(1.6,2.6)	3.6(3,4.5)	3.3(2.5,4.1)
Tianjin	8.1(6.9,9.4)	27.6(26,29.4)	42.7(38.5,46.9)		13.8(12.3,15.4)	17.1(15.8,18.5)	14.8(12.3,17.3)		1.8(1.3,2.4)	7.9(7,8.9)	8.2(6.6,9.8)
Hebei	12.7(11.5,14)	21.6(20.1,23.2)	26.2(23.3,29.1)		19.4(17.9,21)	18.6(17.2,20.1)	18.4(14.7,22.1)		3.7(3.1,4.4)	5.8(5,6.7)	6.3(5.3,7.3)
Shanxi	5.9(5.2,6.8)	15.6(14.3,16.9)	21.8(18.4,25.2)		10.2(9.2,11.3)	12.6(11.5,13.9)	10.9(7.6,14.2)		1.1(0.8,1.5)	3.4(2.8,4.1)	2.7(1.8,3.6)
Inner Mongolia	5.5(4.8,6.4)	18.7(17.4,20.2)	33.6(29.9,37.3)		14.1(12.9,15.4)	15.8(14.6,17.2)	18(14.1,21.9)		1.4(1.1,1.9)	4.8(4.1,5.6)	6.3(5.1,7.5)
Liaoning	8.4(7.7,9.2)	21.6(20,23.2)	29.3(26.2,32.4)		17(16,18)	30.5(28.6,32.4)	31.9(28.2,35.6)		2.4(2,2.8)	9.3(8.3,10.5)	9.9(8.2,11.6)
Jilin	6.4(5.6,7.3)	20(18.4,21.8)	32.9(28.7,37.1)		15.2(14,16.5)	19.1(17.4,20.9)	12.6(9.1,16.1)		1.5(1.1,1.9)	5.2(4.4,6.2)	4.2(3,5.4)
Heilongjiang	7(6.2,8)	24.3(22.6,26)	36.3(32.1,40.5)		14.6(13.4,15.9)	14.6(13.3,15.9)	11.9(9.5,14.3)		1.4(1,1.8)	5.2(4.5,6.1)	5(3.9,6.1)
Shanghai	8.2(7.6,8.9)	17.6(16.2,19)	24.4(20.9,27.9)		7.7(7.1,8.4)	12.5(11.4,13.7)	11.9(9.2,14.6)		1.1(0.9,1.4)	4(3.4,4.8)	3.6(2.4,4.8)
Jiangsu	6.6(5.8,7.5)	12.3(11.2,13.5)	15(12.1,17.9)		4.2(3.6,4.9)	11(10,12.1)	11.7(7,16.4)		0.4(0.2,0.7)	2.1(1.7,2.7)	1.7(0.9,2.5)
Zhejiang	6.6(5.8,7.6)	11.3(10.3,12.5)	13.7(11.1,16.3)		2.7(2.1,3.2)	10.7(9.6,11.8)	20.4(12.4,28.4)		0.4(0.2,0.7)	1.9(1.5,2.4)	1.8(0.9,2.7)
Anhui	3.9(3.3,4.6)	16.9(15.6,18.3)	26.7(22.6,30.8)		8.6(7.6,9.6)	22(20.6,23.6)	27.1(21,33.2)		0.8(0.5,1.1)	4.7(4,5.5)	5.3(3.3,7.3)
Fujian	5.8(5.1,6.7)	14.9(13.7,16.2)	17.8(13.2,22.4)		5.2(4.5,6)	6.6(5.8,7.5)	6.2(1.3,11.1)		0.6(0.4,0.9)	1.9(1.5,2.4)	1.2(-0.9,3.3)
Jiangxi	2.2(1.7,2.7)	11.9(10.7,13.1)	22.6(18.2,27)		6.1(5.3,6.9)	10.6(9.5,11.8)	12.6(6.7,18.5)		0.2(0.1,0.5)	2.3(1.8,2.9)	2.9(0.6,5.2)
Shandong	11(10,12)	28.6(26.9,30.3)	41.5(37.9,45.1)		20.2(18.8,21.6)	22.1(20.7,23.7)	20.6(17,24.2)		4(3.4,4.7)	7.8(7,8.8)	6.7(5.3,8.1)
Henan	5.7(5,6.6)	17(15.6,18.6)	28(23.6,32.4)		16.8(15.4,18.2)	8.6(7.6,9.7)	6.3(4.8,7.8)		2(1.6,2.5)	2.7(2.2,3.4)	2.5(1.9,3.1)
Hubei	1.9(1.5,2.5)	15.3(14.1,16.7)	29.7(25.2,34.2)		7.8(6.9,8.7)	18.2(16.9,19.7)	19.7(13.5,25.9)		0.2(0.1,0.5)	4.1(3.5,4.8)	5.8(3.3,8.3)
Hunan	3.9(3.2,4.6)	16(14.7,17.3)	29.6(24.9,34.3)		6.5(5.7,7.4)	13.2(12.1,14.4)	12.6(7.6,17.6)		0.4(0.2,0.6)	3.2(2.7,3.9)	3.5(1.1,5.9)
Guangdong	3.6(2.9,4.5)	7.9(7,8.8)	10.3(7.2,13.4)		5.1(4.3,6.1)	7.5(6.6,8.4)	7.7(0.7,14.7)		0.6(0.3,1)	1.4(1.1,1.9)	0.9(-0.1,1.9)
Guangxi	4.5(3.8,5.2)	11(9.9,12.2)	18.9(14.9,22.9)		7.6(6.7,8.6)	6.4(5.6,7.3)	7.8(4,11.6)		0.7(0.5,1)	0.9(0.7,1.3)	1.2(0.7,1.7)
Hainan	3.8(3.1,4.6)	13.5(12.3,14.8)	18.5(13.9,23.1)		8.2(7.2,9.3)	8.5(7.6,9.6)	17.5(8.3,26.7)		0.8(0.5,1.2)	1.5(1.1,1.9)	1.3(0.5,2.1)
Chongqing	2.4(1.9,3)	13.7(12.3,15.1)	26.4(19.3,33.5)		7.9(7,8.9)	6(5.1,7)	6.8(1.5,12.1)		0.5(0.3,0.8)	0.7(0.4,1.2)	0.6(0.3,0.9)
Sichuan	3.3(2.8,4)	11.2(10.1,12.3)	17.2(12.9,21.5)		13.4(12.3,14.7)	7.3(6.5,8.3)	7.8(3.2,12.4)		0.8(0.5,1.1)	2(1.6,2.5)	1.6(0.3,2.9)
Guizhou	2.1(1.6,2.6)	13.6(12.5,14.9)	28.6(23.6,33.6)		7.3(6.4,8.2)	10.9(9.8,12)	7.6(2.6,12.6)		0.4(0.2,0.6)	2.5(2,3.1)	2.4(0.7,4.1)

Yunnan	4.4(3.6,5.3)	10.7(9.7,11.7)	17(13.2,20.8)		17.9(16.2,19.6)	10.3(9.3,11.3)	6.5(3,10)		1.3(0.9,1.9)	1.7(1.3,2.2)	1.4(0.5,2.3)
Tibet	/	9.4(8.5,10.3)	13.5(9.6,17.4)		/	21.7(20.4,23.1)	20.1(13,27.2)		/	2.8(2.4,3.3)	2.9(1.7,4.1)
Shaanxi	3.3(2.7,3.9)	16.7(15.4,18)	37.4(31.8,43)		12(10.9,13.2)	8.4(7.6,9.4)	4.6(2.9,6.3)		0.7(0.5,1.1)	2.2(1.7,2.7)	1.7(0.9,2.5)
Gansu	3.5(2.9,4.2)	17.2(15.8,18.7)	32.5(27.9,37.1)		4.3(3.7,5)	13.7(12.5,15)	14.5(9.9,19.1)		0.5(0.3,0.8)	3.4(2.8,4.1)	2(0.6,3.4)
Qinghai	1.8(1.1,2.7)	13.4(12.1,14.9)	28.2(22.4,34)		13.4(11.4,15.7)	23.8(22,25.7)	19.7(12.9,26.5)		0.5(0.2,1.1)	4.5(3.7,5.3)	4.2(2.6,5.8)
Ningxia	2.8(2.3,3.4)	13.9(12.5,15.5)	23.1(18.8,27.4)		19.7(18.3,21.3)	17.1(15.6,18.9)	12.3(7.9,16.7)		1(0.7,1.3)	3.4(2.7,4.2)	2.6(1.2,4)
Xinjiang	2.9(2,4.1)	16.9(15.6,18.3)	36.6(29.9,43.3)		8.7(7.2,10.6)	9.7(8.7,10.8)	8.1(5.7,10.5)		1(0.5,1.7)	3.3(2.7,4)	3.4(2.1,4.7)
**Both sexes**											
Beijing	7.3(6.2,8.8)	26.1(24.9,27.4)	34.5(30.9,38.1)		10.9(10.1,11.7)	13.2(12.2,14.2)	14.2(12.7,15.7)		3.3(2.9,3.7)	6.2(5.6,6.9)	7.3(6.4,8.2)
Tianjin	15.5(13.5,17.8)	34.5(33.1,35.9)	51.9(48.5,55.3)		16.3(15.1,17.6)	21.9(20.8,23)	22.7(20.9,24.5)		2.1(1.7,2.6)	11.2(10.5,12)	15.5(14,17)
Hebei	9.7(8.3,11.3)	28.5(27.2,29.8)	35.5(32.9,38.1)		23.2(22,24.4)	23.8(22.7,25)	23.5(21.1,25.9)		5.5(5,6.2)	8.3(7.6,9)	9.7(8.7,10.7)
Shanxi	5.2(4.2,6.6)	20.7(19.6,21.8)	28.9(26,31.8)		11(10.2,11.8)	16.6(15.7,17.6)	15.8(13.4,18.2)		2.1(1.8,2.5)	5.4(4.9,6)	5.2(4.3,6.1)
Inner Mongolia	10.9(8.9,13.4)	25.3(24.2,26.5)	43.4(40.4,46.4)		18.1(17.1,19.2)	20.8(19.7,21.8)	22.1(19.7,24.5)		2.3(2,2.8)	8(7.4,8.7)	10.9(9.7,12.1)
Liaoning	18.5(15.6,21.9)	29.7(28.3,31)	41.5(39.1,43.9)		20(19.2,20.8)	34.3(32.9,35.8)	36.5(33.8,39.2)		3.6(3.3,4)	14(13.1,14.9)	18.5(17,20)
Jilin	8.5(6.9,10.6)	25.3(24.1,26.7)	41.2(37.9,44.5)		15.8(14.9,16.7)	23.1(21.9,24.5)	20(17.5,22.5)		1.8(1.5,2.1)	7.4(6.7,8.2)	8.5(7.5,9.5)
Heilongjiang	11.1(9.6,12.9)	32.5(31.2,33.9)	45.7(42.4,49)		18.8(17.8,19.8)	18.9(17.9,20)	18.9(17.2,20.6)		2.5(2.1,2.9)	8.7(8,9.4)	11.1(10,12.2)
Shanghai	9(7.2,11.9)	25(23.9,26.3)	36(32.9,39.1)		10.8(10.2,11.3)	17.9(16.9,18.9)	18(16.1,19.9)		2.2(2,2.5)	7.4(6.7,8)	9(7.9,10.1)
Jiangsu	3.7(2.8,5.2)	18.1(17.1,19.2)	20.5(17.5,23.5)		6.1(5.5,6.7)	15.2(14.3,16.1)	17.1(14.1,20.1)		1.1(0.8,1.3)	4.2(3.7,4.7)	3.7(2.9,4.5)
Zhejiang	3.7(2.7,5)	17.6(16.6,18.6)	22.6(20,25.2)		4.2(3.7,4.7)	13.5(12.7,14.4)	22.5(18.3,26.7)		0.7(0.5,0.9)	3.6(3.2,4.1)	3.7(2.9,4.5)
Anhui	13.3(10.6,16.7)	24.1(23,25.2)	39.6(36.6,42.6)		12(11.2,12.8)	26.4(25.2,27.6)	33.7(29.7,37.7)		1.4(1.1,1.7)	8.2(7.6,8.9)	13.3(11.7,14.9)
Fujian	3.2(2.1,6.5)	22.7(21.6,23.8)	27.8(24.1,31.5)		7.7(7.1,8.4)	9.9(9.2,10.7)	9.4(6.7,12.1)		1.5(1.2,1.8)	4(3.6,4.5)	3.2(1.9,4.5)
Jiangxi	4.9(3.5,8.4)	17.8(16.8,18.9)	32.4(28.9,35.9)		7.3(6.7,8)	14.8(13.9,15.8)	17.4(13.8,21)		0.8(0.6,1)	4.3(3.8,4.8)	4.9(3.5,6.3)
Shandong	12.3(10.9,14)	37.5(36.1,38.9)	50.2(47.2,53.2)		24(23,25.1)	26.8(25.7,28)	25.4(23.3,27.5)		6.9(6.3,7.5)	11.7(11,12.5)	12.3(11.2,13.4)
Henan	5.4(4.4,6.6)	23.3(22,24.5)	35.1(31.6,38.6)		18.4(17.4,19.4)	12.7(11.8,13.6)	10.3(8.9,11.7)		2.4(2.1,2.8)	5(4.4,5.6)	5.4(4.6,6.2)
Hubei	10.8(8.3,14)	21.7(20.6,22.8)	41.5(38.2,44.8)		10.9(10.2,11.7)	23.2(22.1,24.4)	25.3(21.7,28.9)		0.6(0.4,0.8)	7(6.4,7.7)	10.8(9,12.6)
Hunan	3.9(2.8,6.3)	21.7(20.7,22.9)	36.4(32.6,40.2)		7.4(6.8,8.1)	15.7(14.8,16.6)	14.2(11.2,17.2)		1(0.7,1.2)	4.4(4,4.9)	3.9(2.6,5.2)
Guangdong	1.6(1,3.1)	11.7(11,12.5)	18.3(15.6,21)		6.8(6.1,7.6)	9.7(9,10.4)	10.9(6.6,15.2)		0.6(0.4,0.9)	2.2(1.9,2.6)	1.6(0.7,2.5)
Guangxi	2.6(1.9,3.7)	16.2(15.3,17.2)	27.4(24,30.8)		7.6(7,8.3)	8.5(7.8,9.2)	9.5(7.2,11.8)		1.2(0.9,1.5)	2.2(1.9,2.6)	2.6(2,3.2)
Hainan	2.7(2.1,3.6)	17.9(17,19)	27(23.9,30.1)		10.9(10.1,11.8)	9.6(8.9,10.3)	15.7(10.9,20.5)		1.1(0.8,1.3)	2.7(2.3,3.1)	2.7(2.1,3.3)
Chongqing	1.2(0.8,1.8)	18(17,19.2)	34.5(29.3,39.7)		8.7(8.1,9.5)	8(7.3,8.8)	7.5(4.7,10.3)		0.8(0.6,1)	1.6(1.3,2)	1.2(0.8,1.6)
Sichuan	2(1.3,5.5)	14.8(13.9,15.7)	23.3(19.7,26.9)		13.4(12.6,14.3)	10.1(9.3,10.8)	10.7(7.8,13.6)		1.3(1,1.6)	2.8(2.5,3.2)	2(1.2,2.8)

Guizhou	2.9(1.7,8.1)	16.6(15.6,17.6)	32.5(28.3,36.7)		7.6(7,8.3)	12.8(12,13.7)	12.3(9.1,15.5)		0.5(0.4,0.7)	3.1(2.7,3.6)	2.9(1.7,4.1)
Yunnan	2.7(1.9,3.9)	16.1(15.2,17)	26.4(23.2,29.6)		18.6(17.4,19.9)	13.5(12.7,14.3)	10.3(7.9,12.7)		2(1.6,2.4)	3.1(2.8,3.6)	2.7(2,3.4)
Tibet	/	9.9(9.3,10.6)	13.2(10.8,15.6)		/	22.4(21.5,23.4)	23.5(17.6,29.4)		/	3.1(2.8,3.5)	3.8(3,4.6)
Shaanxi	4(2.9,5.5)	22.5(21.5,23.6)	43.9(39.8,48)		13.4(12.5,14.3)	11.4(10.6,12.2)	8.6(7.3,9.9)		1.4(1.1,1.7)	3.8(3.4,4.3)	4(3.2,4.8)
Gansu	4.1(3,6.2)	20.9(19.8,22)	36(32.5,39.5)		6.1(5.5,6.6)	17(16.1,18)	19.5(16.5,22.5)		0.8(0.6,1)	4.7(4.2,5.3)	4.1(3.2,5)
Qinghai	6(4.3,8.6)	17.8(16.7,19)	31.8(27.8,35.8)		15.8(14.2,17.4)	26.1(24.7,27.5)	20.5(16.2,24.8)		1(0.6,1.5)	6.9(6.2,7.6)	6(4.2,7.8)
Ningxia	7.4(5.4,10.6)	19.4(18.3,20.7)	34.3(31,37.6)		21.3(20.3,22.5)	21.1(19.8,22.4)	19.6(16.9,22.3)		1.6(1.4,2)	5.5(4.9,6.2)	7.4(6.2,8.6)
Xinjiang	7.9(5.9,10.5)	23.7(22.5,24.8)	44.5(39.4,49.6)		11(9.7,12.4)	14.9(14,15.9)	15.7(13.8,17.6)		1.3(0.8,1.8)	6(5.5,6.7)	7.9(6.7,9.1)

Table S6. Chinese demographic trends in 2000, 2019, and 2030 at the national and provincial level

	2000					2019					2030				
	Share of population aged 0-14 (%)	Birth rate (‰)	Share of population aged 65+ (%)	Death rate (‰)	Population development index	Share of population aged 0-14 (%)	Birth rate (‰)	Share of population aged 65+ (%)	Death rate (‰)	Population development index	Share of population aged 0-14 (%)	Birth rate (‰)	Share of population aged 65+ (%)	Death rate (‰)	Population development index
Beijing	14.96 (30)	6.5 (30)	10.27 (2)	5.6 (27)	0.53 (30)	10.42 (27)	8.12 (26)	11.45 (19)	5.49 (27)	0.3 (25)	11.35 (23)	5.1 (25)	20.75 (12)	6.25 (26)	-0.81 (21)
Tianjin	18.63 (27)	9.68 (29)	9.36 (5)	6.73 (13)	1.05 (29)	10.28 (28)	6.73 (28)	12.08 (14)	5.3 (28)	0.08 (27)	10.51 (27)	4.8 (27)	23.07 (5)	5.45 (28)	-0.91 (24)
Hebei	24.19 (18)	12.99 (17)	6.6 (22)	6.26 (22)	2.03 (15)	18.83 (11)	10.83 (15)	13.14 (11)	6.12 (16)	0.93 (14)	13.43 (16)	6.78 (17)	20.09 (13)	8 (9)	-0.57 (17)
Shanxi	26.61 (11)	15.93 (9)	6.41 (23)	6.07 (24)	2.39 (8)	15.37 (21)	9.12 (22)	10.99 (20)	5.85 (22)	0.78 (16)	12.92 (19)	6.74 (19)	19.95 (15)	6.83 (21)	-0.45 (15)
Inner Mongolia	22.21 (22)	13.32 (16)	5.85 (25)	6.08 (23)	2.12 (12)	13.03 (25)	8.23 (24)	10.2 (21)	5.66 (24)	0.62 (20)	11.27 (24)	5.59 (23)	21.24 (9)	7.69 (13)	-0.95 (25)
Liaoning	18.48 (29)	10.38 (28)	7.81 (13)	7.05 (5)	1.25 (26)	10.2 (29)	6.45 (29)	15.92 (2)	7.25 (4)	-0.56 (31)	8.68 (29)	4.13 (28)	27.23 (1)	10.36 (1)	-2.06 (30)
Jilin	18.59 (28)	10.68 (24)	6.78 (19)	5.45 (29)	1.68 (22)	11.77 (26)	6.05 (30)	13.29 (10)	6.9 (9)	-0.25 (29)	8.28 (30)	4.11 (29)	25.47 (3)	7.51 (14)	-1.73 (29)
Heilongjiang	20.62 (24)	10.55 (26)	5.42 (28)	5.49 (28)	1.99 (17)	9.97 (31)	5.73 (31)	13.78 (9)	6.74 (12)	-0.49 (30)	6.96 (31)	3.52 (31)	25.74 (2)	7.89 (11)	-2.11 (31)
Shanghai	13.37 (31)	5.4 (31)	13.84 (1)	6.5 (14)	-0.22 (31)	10.03 (30)	7 (27)	16.27 (1)	5.5 (26)	-0.24 (28)	8.79 (28)	3.86 (30)	23.91 (4)	7.1 (18)	-1.61 (28)
Jiangsu	19.99 (25)	10.5 (27)	9.83 (3)	6.94 (9)	1.12 (28)	13.79 (23)	9.12 (23)	15.08 (6)	7.04 (7)	0.17 (26)	10.88 (26)	4.92 (26)	22.28 (6)	8.47 (7)	-1.26 (27)
Zhejiang	18.98 (26)	10.64 (25)	9.54 (4)	6.35 (19)	1.2 (27)	13.06 (24)	10.51 (19)	14.03 (7)	5.52 (25)	0.57 (21)	11.05 (25)	5.24 (24)	19.91 (16)	6.79 (23)	-0.85 (22)
Anhui	25.23 (16)	15.1 (12)	7.43 (14)	6.5 (15)	2.07 (14)	18.65 (12)	12.03 (11)	13.97 (8)	6.04 (20)	0.98 (13)	14.28 (14)	7.32 (12)	19.08 (18)	7.83 (12)	-0.36 (14)
Fujian	25.72 (15)	11.06 (23)	8.04 (9)	5.85 (25)	1.8 (20)	16.87 (16)	12.9 (6)	10 (24)	6.1 (18)	1.27 (11)	14.57 (12)	7.06 (14)	16.26 (22)	6.81 (22)	-0.07 (13)
Jiangxi	26.59 (12)	16.51 (8)	6.71 (20)	7.02 (7)	2.23 (11)	20.21 (7)	12.59 (9)	10.15 (23)	6.03 (21)	1.42 (8)	14.63 (11)	8.36 (9)	16.92 (20)	7.03 (19)	0.03 (11)
Shandong	21.43 (23)	11.08 (22)	8.61 (7)	6.27 (21)	1.48 (25)	17.67 (14)	11.77 (12)	15.84 (3)	7.5 (2)	0.56 (22)	14.46 (13)	6.99 (15)	21.08 (11)	8.56 (5)	-0.58 (18)
Henan	26.14 (14)	14.07 (14)	7.08 (17)	6.35 (20)	2.1 (13)	21.06 (5)	11.02 (14)	11.6 (16)	6.84 (10)	1.07 (12)	15.15 (10)	8.17 (10)	18.11 (19)	7.12 (17)	-0.04 (12)
Hubei	25.17 (17)	11.57 (21)	7.15 (15)	6.37 (18)	1.86 (19)	15.57 (20)	11.35 (13)	13.07 (13)	7.08 (6)	0.65 (19)	12.26 (22)	5.62 (22)	21.18 (10)	9.27 (4)	-1.05 (26)
Hunan	23.19 (20)	11.72 (20)	7.98 (10)	7.12 (4)	1.56 (23)	19.99 (8)	10.39 (21)	13.14 (12)	7.28 (3)	0.77 (17)	13.57 (15)	7.14 (13)	19.62 (17)	9.76 (2)	-0.68 (19)
Guangdong	27.79 (5)	15.32 (11)	7.89 (12)	5.4 (30)	2.3 (10)	15.97 (19)	12.54 (10)	8.58 (28)	4.46 (29)	1.65 (4)	15.69 (9)	8.02 (11)	12.82 (28)	4.83 (29)	0.71 (4)
Guangxi	27.2 (9)	14.96 (13)	7.96 (11)	6.93 (11)	2 (16)	21.83 (4)	13.31 (5)	10.17 (22)	6.14 (15)	1.54 (6)	18.16 (3)	10.85 (3)	16.22 (23)	7.19 (16)	0.52 (7)
Hainan	28.5 (3)	17.26 (7)	6.63 (21)	5.23 (31)	2.65 (5)	19.51 (10)	12.87 (7)	9.32 (27)	6.11 (17)	1.48 (7)	15.75 (8)	8.61 (8)	15.96 (24)	2.93 (31)	1.07 (2)
Chongqing	23.07 (21)	11.9 (19)	8.84 (6)	6.94 (10)	1.5 (24)	16.78 (17)	10.48 (20)	15.35 (5)	7.57 (1)	0.41 (24)	12.39 (21)	6.79 (16)	21.44 (8)	9.63 (3)	-0.9 (23)
Sichuan	23.54 (19)	13.8 (15)	8.33 (8)	7.02 (6)	1.71 (21)	16.41 (18)	10.7 (16)	15.74 (4)	7.09 (5)	0.45 (23)	12.43 (20)	6.75 (18)	21.63 (7)	8.37 (8)	-0.77 (20)
Guizhou	28.13 (4)	21.92 (2)	6.05 (24)	7.68 (2)	2.59 (6)	22.25 (3)	13.65 (4)	11.58 (17)	6.95 (8)	1.33 (9)	20.65 (2)	13.3 (1)	14.23 (26)	7.41 (15)	0.96 (3)
Yunnan	27.51 (6)	19.48 (4)	6.83 (18)	7.82 (1)	2.31 (9)	18.04 (13)	12.63 (8)	9.88 (25)	6.2 (14)	1.31 (10)	16.51 (6)	9.7 (6)	14.82 (25)	7.89 (10)	0.31 (8)
Tibet	35.14 (1)	23.2 (1)	4.08 (31)	7.4 (3)	3.29 (1)	26.07 (1)	14.6 (1)	6.01 (31)	4.46 (30)	2.65 (1)	20.78 (1)	12.56 (2)	8.92 (31)	4.66 (30)	1.84 (1)
Shaanxi	26.28 (13)	12.51 (18)	7.14 (16)	6.38 (17)	1.98 (18)	14.55 (22)	10.55 (18)	12.05 (15)	6.28 (13)	0.71 (18)	13.25 (17)	6.02 (21)	20.08 (14)	6.65 (24)	-0.51 (16)
Gansu	27.33 (8)	15.61 (10)	5.6 (26)	6.44 (16)	2.47 (7)	17.1 (15)	10.6 (17)	11.51 (18)	6.75 (11)	0.85 (15)	16.39 (7)	9.18 (7)	16.9 (21)	8.55 (6)	0.04 (10)
Qinghai	27.43 (7)	20.68 (3)	5.46 (27)	6.78 (12)	2.73 (4)	19.52 (9)	13.66 (3)	8.53 (29)	6.08 (19)	1.64 (5)	16.78 (5)	9.73 (5)	12.7 (29)	6.37 (25)	0.7 (5)
Ningxia	28.75 (2)	17.97 (6)	4.47 (30)	5.65 (26)	3.02 (2)	20.73 (6)	13.72 (2)	9.5 (26)	5.69 (23)	1.66 (3)	17.43 (4)	10.11 (4)	13.29 (27)	6.9 (20)	0.65 (6)
Xinjiang	27.11 (10)	18.76 (5)	4.69 (29)	6.96 (8)	2.75 (3)	24.14 (2)	8.14 (25)	8.07 (30)	4.45 (31)	1.7 (2)	13.14 (18)	6.45 (20)	12.16 (30)	6.08 (27)	0.14 (9)
China	23.94	15.23	7.63	6.46	2.00	16.78	10.48	12.57	7.14	0.67	13.43	6.79	19.08	7.68	-0.47

Note: The Population Development Index is calculated by dividing the birth rate by the death rate multiplied by the share of the population aged 0-14 years divided by the share of the population aged over 65 years and then taking their natural log value. The rank was presented in parentheses.

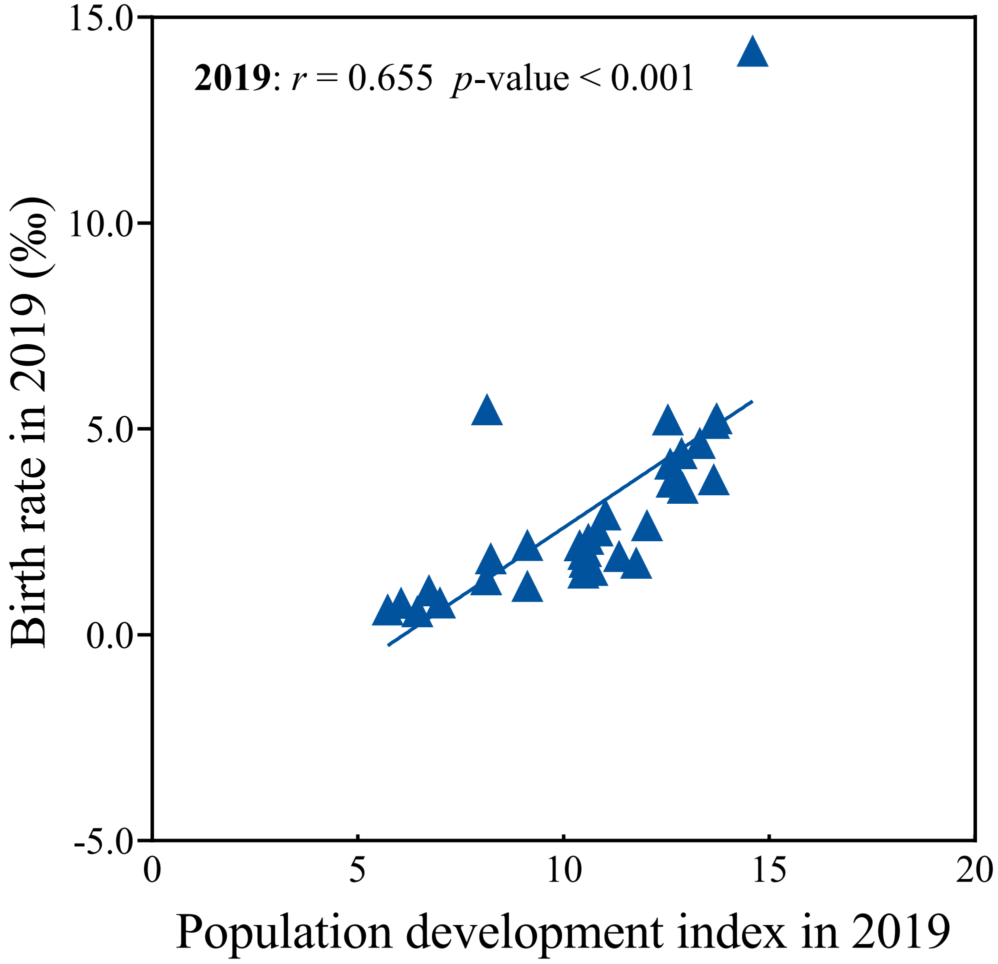

Figure S1. The association between the Population Development Index and the birth rate in 2019

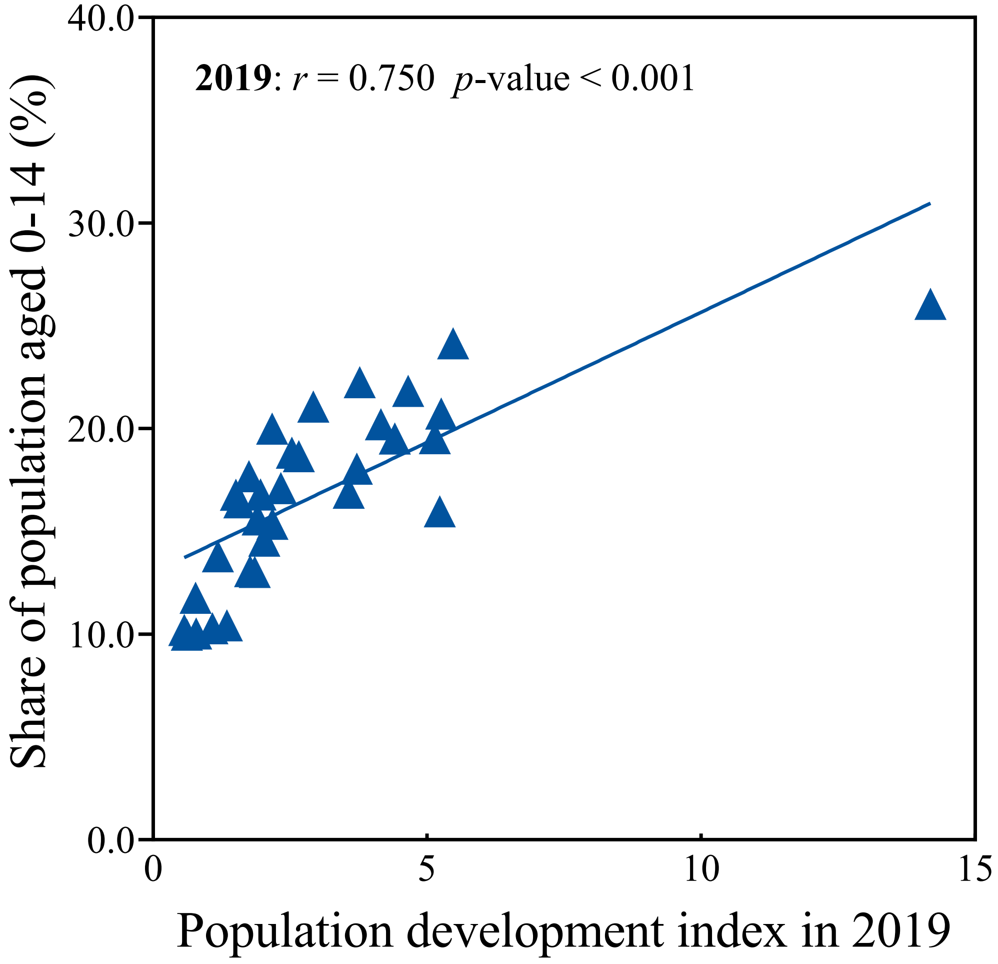

Figure S2. The association between the Population Development Index and the share of population aged 0-14 in 2019

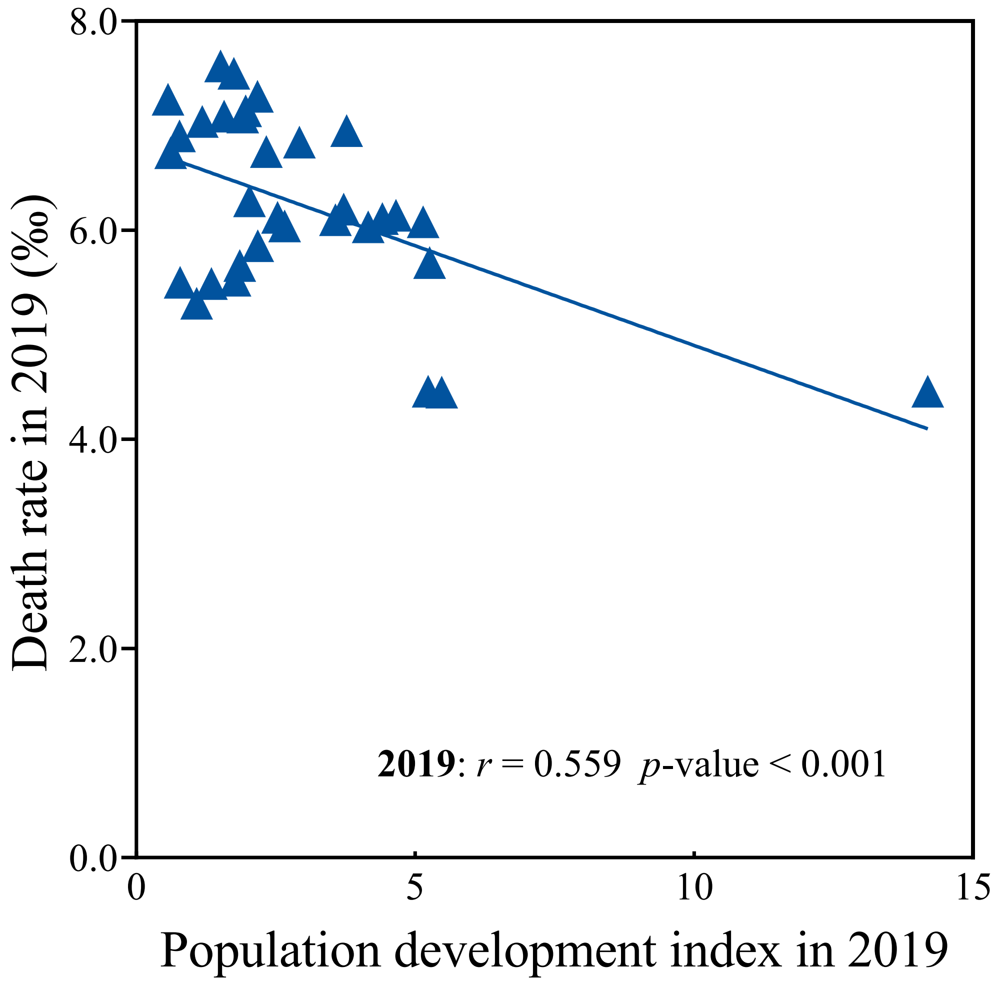

Figure S3. The association between the Population Development Index and the death rate in 2019

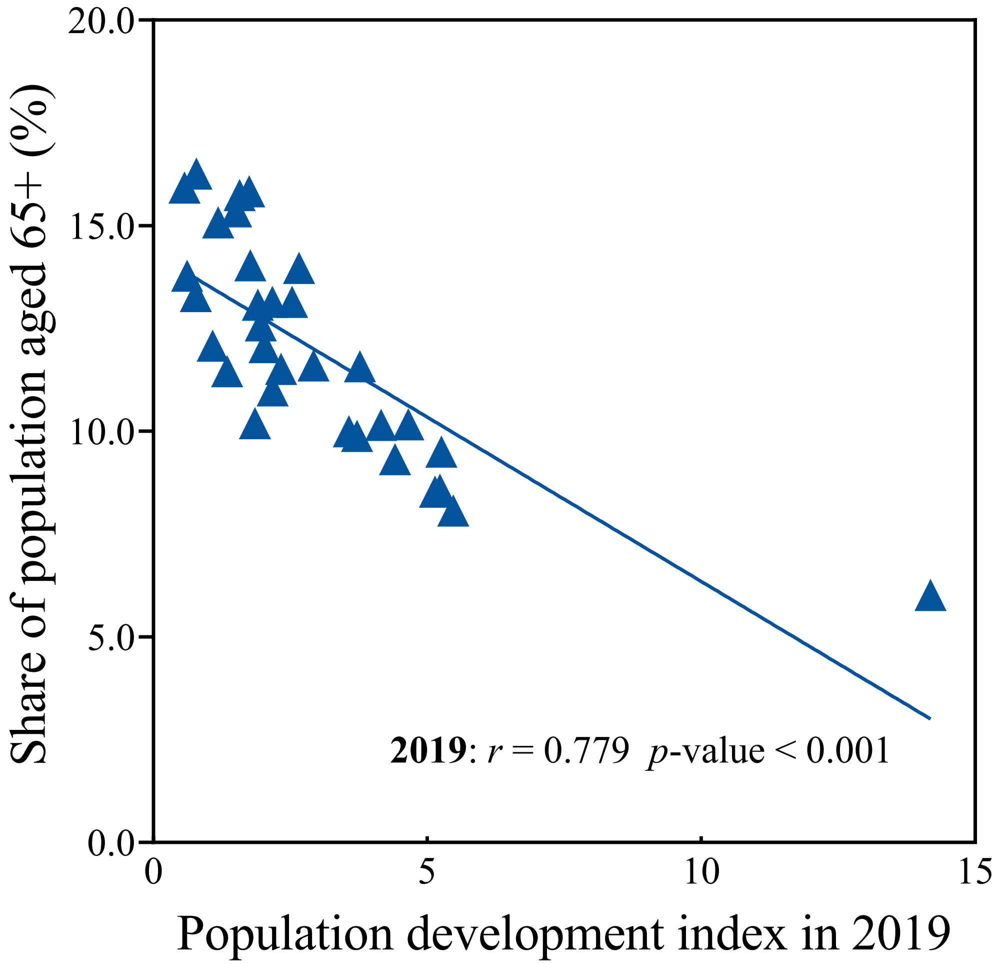

Figure S4. The association between the Population Development Index and the share of population aged 65+ (%) in 2019

Table S8. Predicted estimates of sex-specific and age-specific OWOB prevalence among Chinese children and adolescents aged 7–18 years in 2030

	Bootstrap Method				Maximum Likelihood Estimation			
	lower_ci	upper_ci	variance	se	lower_ci	upper_ci	variance	se
Boy								
7	0.4187	0.4544	0.0001	0.0092	0.3975	0.4731	0.0004	0.0193
8	0.4471	0.4821	0.0001	0.0096	0.4273	0.4988	0.0003	0.0182
9	0.4957	0.5309	0.0001	0.0087	0.4794	0.5507	0.0003	0.0182
10	0.5158	0.5459	0.0001	0.0078	0.4946	0.5647	0.0003	0.0179
11	0.5234	0.5524	0.0001	0.0079	0.5056	0.5714	0.0003	0.0168
12	0.4630	0.4989	0.0001	0.0087	0.4532	0.5104	0.0002	0.0146
13	0.4205	0.4566	0.0001	0.0101	0.4136	0.4647	0.0002	0.0130
14	0.3846	0.4177	0.0001	0.0092	0.3845	0.4246	0.0001	0.0102
15	0.3270	0.3585	0.0001	0.0093	0.3266	0.3612	0.0001	0.0088
16	0.3134	0.3543	0.0001	0.0100	0.3172	0.3514	0.0001	0.0087
17	0.2932	0.3342	0.0001	0.0112	0.2979	0.3304	0.0001	0.0083
18	0.2576	0.2906	0.0001	0.0094	0.2588	0.2916	0.0001	0.0084
Girl								
7	0.2499	0.2913	0.0001	0.0098	0.2461	0.2995	0.0002	0.0136
8	0.2822	0.3182	0.0001	0.0099	0.2735	0.3278	0.0002	0.0139
9	0.3077	0.3495	0.0001	0.0102	0.3027	0.3564	0.0002	0.0137
10	0.3161	0.3492	0.0001	0.0095	0.3077	0.3608	0.0002	0.0135
11	0.3194	0.3528	0.0001	0.0096	0.3131	0.3628	0.0002	0.0127
12	0.3039	0.3455	0.0001	0.0110	0.2986	0.3459	0.0001	0.0121
13	0.2987	0.3358	0.0001	0.0095	0.2934	0.3420	0.0002	0.0124
14	0.2943	0.3319	0.0001	0.0093	0.2912	0.3354	0.0001	0.0113
15	0.2103	0.2513	0.0001	0.0096	0.2101	0.2490	0.0001	0.0099
16	0.2019	0.2400	0.0001	0.0097	0.1984	0.2355	0.0001	0.0095
17	0.1703	0.2130	0.0001	0.0109	0.1714	0.2053	0.0001	0.0086
18	0.1186	0.1541	0.0001	0.0088	0.1216	0.1502	0.0001	0.0073

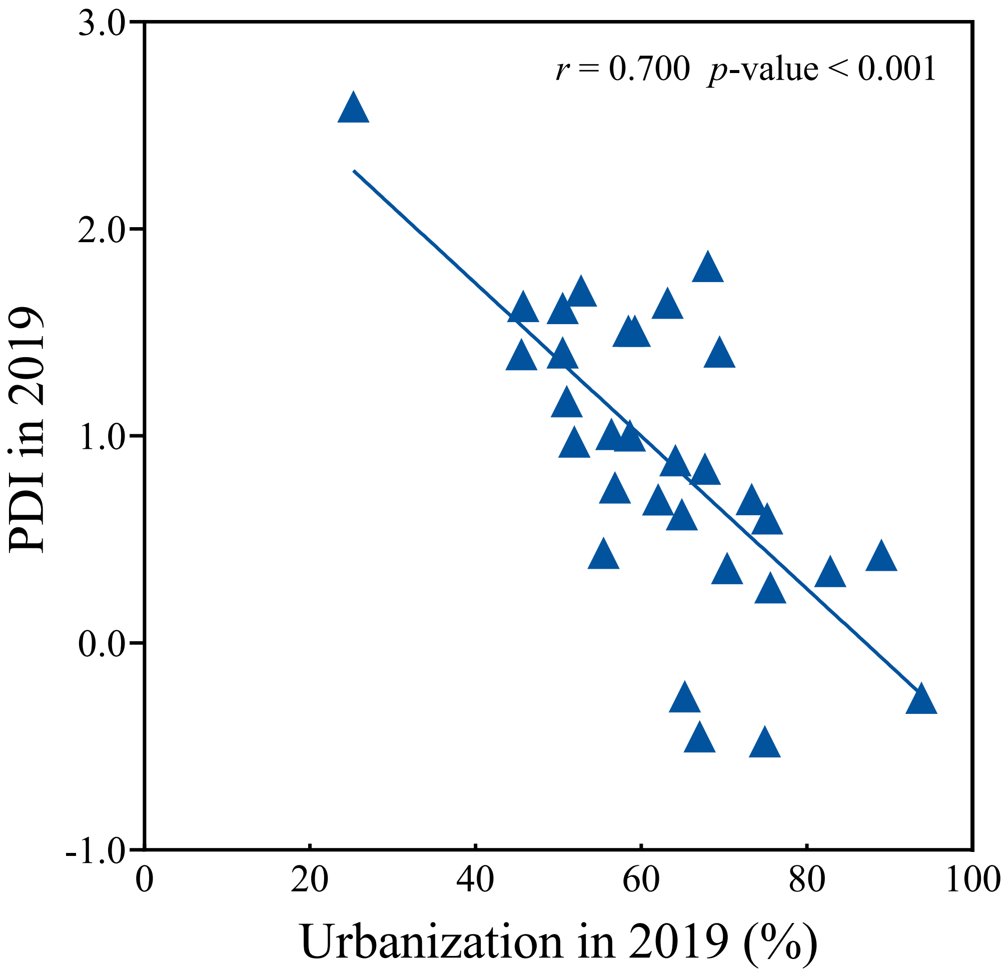

Figure S5. The association between urbanization rate and the Population Development Index in 2019
